# Molecular Signaling Regulating Endometrium–Blastocyst Crosstalk

**DOI:** 10.3390/ijms21010023

**Published:** 2019-12-18

**Authors:** Micol Massimiani, Valentina Lacconi, Fabio La Civita, Carlo Ticconi, Rocco Rago, Luisa Campagnolo

**Affiliations:** 1 Department of Biomedicine and Prevention, University of Rome Tor Vergata, Via Montpellier 1, 00133 Rome, Italy; micol.massimiani@unicamillus.org (M.M.); valelcc@gmail.com (V.L.); fabio.lacivita25@gmail.com (F.L.C.); 2 Saint Camillus International University of Health Sciences, Via di Sant’Alessandro, 8, 00131 Rome, Italy; 3 Department of Surgical Sciences, Section of Gynecology and Obstetrics, University Tor Vergata, Via Montpellier, 1, 00133 Rome, Italy; ticconi@uniroma2.it; 4 Physiopathology of Reproduction and Andrology Unit, Sandro Pertini Hospital, Via dei Monti Tiburtini 385/389, 00157 Rome, Italy; rocco.rago@aslroma2.it

**Keywords:** implantation, endometrium, blastocyst, embryo, chorionic gonadotropin, progesterone, Notch, cytokines

## Abstract

Implantation of the embryo into the uterine endometrium is one of the most finely-regulated processes that leads to the establishment of a successful pregnancy. A plethora of factors are released in a time-specific fashion to synchronize the differentiation program of both the embryo and the endometrium. Indeed, blastocyst implantation in the uterus occurs in a limited time frame called the “window of implantation” (WOI), during which the maternal endometrium undergoes dramatic changes, collectively called “decidualization”. Decidualization is guided not just by maternal factors (e.g., estrogen, progesterone, thyroid hormone), but also by molecules secreted by the embryo, such as chorionic gonadotropin (CG) and interleukin-1β (IL-1 β), just to cite few. Once reached the uterine cavity, the embryo orients correctly toward the uterine epithelium, interacts with specialized structures, called pinopodes, and begins the process of adhesion and invasion. All these events are guided by factors secreted by both the endometrium and the embryo, such as leukemia inhibitory factor (LIF), integrins and their ligands, adhesion molecules, Notch family members, and metalloproteinases and their inhibitors. The aim of this review is to give an overview of the factors and mechanisms regulating implantation, with a focus on those involved in the complex crosstalk between the blastocyst and the endometrium.

## 1. Introduction

Implantation requires a complex crosstalk between the endometrium and the blastocyst and is highly regulated by a variety of factors, such as soluble growth factors, hormones, prostaglandins, adhesion molecules, and the extracellular matrix (ECM) [1,2,3,4,5]. These factors, produced by the receptive endometrium in response to the presence of the blastocyst and vice versa, are able to synchronize the development of the embryo to the blastocyst stage and the differentiation of the uterus to the receptive state [6,7]. This complex network of signaling accounts for implantation being one of the major limiting steps in mammalian reproduction. Indeed, the implantation rate in humans is about 30% per cycle [8,9]. Alterations of these signaling pathways may result in pathological conditions leading to infertility.

The WHO has designated infertility as “a disease of the reproductive system defined by the failure to achieve a clinical pregnancy after 12 months or more of regular unprotected sexual intercourse” [10,11]. Infertility is one of the main health issues in all societies worldwide, with a prevalence of 3.5–16.7% in developed countries and 6.9–9.3% in developing countries [12,13] and may be a consequence of low embryo quality, male problems, or female dysfunctions. Female fertility problems account for 20–35% of infertility cases and may derive from a wide variety of causes such as age, anatomical, endocrine and immunological problems, and several pathological conditions affecting the endometrium [14,15,16,17,18,19]. These conditions may lead to defects in blastocyst implantation in the maternal uterus, resulting in implantation failure, a common cause of impaired fertility [20]. The term “implantation failure” actually implies a series of conditions in which the embryo does not implant in the maternal endometrium after both spontaneous and in vitro fertilization (IVF) [21]. A condition in which implantation failure occurs after the transfer of three or more good quality embryos is defined recurrent implantation failure (RIF) and it is only applicable to assisted reproductive technology (ART) [21,22]. According to ASRM and ESHRE definitions, RIF is considered a distinct pathological condition from recurrent pregnancy loss [21,23,24].

The present review describes and discusses the molecular mechanisms underlying the implantation process, focusing on factors implicated in the complex blastocyst–endometrium crosstalk, which are crucial for successful implantation. Further research for new factors involved in the dialogue between the blastocyst and the endometrium would allow to reduce the current rates of implantation failure, allowing many couples with infertility problems to reach a successful pregnancy.

## 2. Preparation of the Endometrium to Implantation

Interaction between the uterus and the blastocyst can only occur during a limited defined period, known as the “window of implantation” (WOI) [25,26,27]. In humans, this defined period corresponds to the mid-secretory phase, occurring between the 20th and the 24th day of the menstrual cycle, or 6-10 days after the luteinizing hormone (LH) peak [25,28,29,30]. In this timeframe, the molecular program regulating growth and differentiation of the embryo synchronizes with the molecular program regulating endometrial receptivity. Failure in such synchronization results in failure of the blastocyst to implant. Given the relevance of this stage for the establishment of a successful pregnancy, the WOI is regulated by a wide variety of cytokines, growth factors, prostaglandins, enzymes, and adhesion molecules [31,32,33].

### 2.1. Gland Development and Function

During the WOI, the uterine endometrium is affected by morphological changes which favor blastocyst implantation [34]. The epithelial cells present vacuoles to a supranuclear position and glands become more irregular with a papillary appearance. Uterine glands are necessary for embryo implantation. Their major development in several mammalian species, including humans, occurs mainly during postnatal life and starts from invagination of the luminal epithelium [35,36,37]. At birth, in humans, glands are sparse, and little deepened into the stroma. At puberty, they extend toward the myometrium and form a coiled network of tubules [36]. Animal studies have demonstrated that progesterone treatment during neonatal life impairs gland development and this severely affects fertility, supporting a central role of endometrial glands for embryo implantation [36]. Experimental data suggest that progesterone treatment may affect the expression of genes central to endometrial adenogenesis, including members of the Wnt family [38], whose expression and adenogenic role has been demonstrated in both glands and stroma [39,40,41,42,43,44,45]. A central role of glands in implantation is also suggested by loss-of-function studies of genes involved in epithelial morphogenesis and proliferation in mice, for example, ablation of the cell–cell adhesion molecule Cdh1 results in epithelial disorganization and absence of glands in the neonatal uterus, with consequent infertility [46]; moreover, conditional knock-out of Sox17 in the uterus is associated with impaired endometrial adenogenesis and infertility [47].

Endometrial glands produce and secrete a cocktail of molecules, the histotroph, including amino acids, glucose and growth factors, which appear to be involved in embryo survival, trophectoderm activation, endometrial invasion and nourishment of the implanted embryo [48,49,50,51,52,53,54,55,56]. Leukemia inhibitory factor (LIF) and vascular endothelial growth factor (VEGF) are produced by uterine glands [57]. Interestingly, several studies have reported differences in composition of the histotroph between fertile and infertile women, strengthening the relevance of gland products in supporting embryo implantation and survival [52,58,59,60,61]. The role of endometrial glands in pregnancy is not limited to implantation. The connection between glands and the intervillous space of the primitive placenta suggests that carbohydrates and lipids produced by the glands may contribute to nurturing the implanted embryo at least until syncytiotrophoblast cells contact the maternal vessels [56]. In addition, growth factors and hormones secreted by the glands during early pregnancy [50] may be involved in placental morphogenesis, considering that receptors for some of these factors have been identified on trophoblast cells [62,63,64,65,66]. Altogether, these data indicate that endometrial glands are central in the establishment of a successful pregnancy and a deeper understanding of their precise role in implantation is of importance to reveal potential causes of infertility, as well as other reproductive disorders.

### 2.2. Decidualization

In addition to the changes occurring in the luminal and glandular epithelium, major changes take place also in the endometrial stroma. The endometrial stromal cells undergo a decidual reaction, in which they proliferate and differentiate from fibroblast-like to epithelial-like cells, which will form the maternal decidua. Decidual cells progressively increase in size and number throughout pregnancy, starting from 9.8% of stromal cells in early pregnancy and arrive to 57.8% at term [67]. The acquisition of the epithelial-like phenotype by stromal cells consists in an increase in size, rounding of the nucleus with increased number of nucleoli, accumulation of glycogen, lipid droplets and secretory granules in the cytoplasm, and expansion of rough endoplasmic reticulum and Golgi apparatus [68]. The term “decidua” derives from Latin “de cadere” and means to fall down, so it refers to the fact that the decidualized uterine tissue is lost after parturition. Decidua is mainly formed by decidualized endometrial stromal cells, but also contains hematopoietic cells, macrophages, uterine natural killer (uNK) and monocytes [69,70]. Decidualization starts in the luteal phase, with stromal cells surrounding the spiral arteries in the upper two-thirds of the endometrium, regardless of whether or not the blastocyst is present [71]. Differently from most mammals, decidualization in humans occurs before the embryo reaches the uterine cavity and is driven by the postovulatory rise in progesterone levels and local increase of cyclic adenosine monophosphate (cAMP) production, occurring long before the embryo is ready to implant. In the absence of pregnancy, progesterone levels decrease, and menstrual shedding and cyclic regeneration of the endometrium occur. Decidualization is responsible for embryo quality control, promoting implantation and development, or facilitating early rejection in case, for example, of chromosomally abnormal human embryos [72,73].

### 2.3. Hormone Signaling

Uterine function is primarily regulated by estrogen and progesterone, which modulate gene expression of luminal and glandular epithelium and stromal cells. These ovarian hormones guide the structural and functional remodeling occurring during decidualization. Estrogen receptor (ER) exists in two forms, ERα and ERβ with distinct biological function, both expressed in the endometrium. ERα is essential for implantation since ERα knockout mice display endometrial hypoplasia and are infertile [74]; mice knockout for ERβ present normal endometrium and appear fertile, suggesting that ERβ may be involved in other aspects of endometrial function [75,76,77]. During the proliferative phase, high levels of estrogen induce proliferation of the epithelial, stromal, and vascular endothelial cells [78,79]. Indeed, activated ERα induces proliferation of human epithelial cells and decidualization in stromal cells through rapid non-genomic activation of the extracellular signal-regulated kinase/mitogen-activated protein kinase (ERK/MAPK) pathway [80,81]. In addition to this rapid activity, estrogen increases epithelial proliferation by inducing insulin-like growth factor 1 (IGF1). IGF1 is expressed and secreted by the stroma, and by binding its receptor IGF1R in the epithelium, induces the phosphoinositide 3-kinase (PI3K)/ serine/threonine protein kinase (AKT) pathway leading to proliferation [82,83,84]. Other known targets of estrogen in the endometrium are fibroblast growth factor (FGF)-9, CCAAT enhancer binding protein beta (C/EBPβ) and Mucin 1 (MUC1). FGF-9 is expressed at high levels in the stromal compartment of the endometrium during the late proliferative phase; in vitro FGF-9 stimulates stromal cell proliferation, and expression of FGF-9 in such cells is induced upon 17β-estradiol stimulation [85], suggesting that in vivo estrogen may induce proliferation of stromal cells through the up-regulation of FGF-9. Estrogen-induced proliferation of endometrial cells is also mediated by C/EBPβ, whose pro-proliferative action is exerted on both the endometrial epithelium and stroma through regulation of cyclin-dependent kinases involved in G2 to M transition of mitosis [86]. Estrogen also regulates the expression of the glycoprotein MUC1, which is expressed on the surface of the luminal epithelium to create a protective barrier that has to be removed at implantation to allow embryo attachment [87]. Beyond its activity on cell proliferation, estrogen also induces endometrial expression of leukemia inhibitory factor (LIF), an interleukin-6 family cytokine whose central role for successful implantation and decidualization has been widely reported [88,89] and discussed later in this review. During the proliferative phase, estrogen induces progesterone receptor (PR) in endometrial cells through ERα to determine progesterone responsiveness during the luteal phase, and in turn PR inhibits ERα expression in a negative feedback crucial for endometrial function [90]. Progesterone increases during the secretory phase of the menstrual cycle, inducing decidualization and thus opening the WOI and remains elevated if pregnancy occurs [91]. The effects of progesterone in endometrial cells are mediated by PR, which exists in two isoforms, PR-A and PR-B, transcribed from two promoters of the same gene. Deletion of either PR-A or PR-B demonstrates specific roles of each PR isoform in mediating progesterone actions on the murine uterus. In PR-B knockout mice, progesterone effects mediated by PR-A are sufficient for a normal uterine function, since implantation, pregnancy, and parturition are normal in these mice [92]. On the contrary, in PR-A-knockout mice progesterone actions mediated by PR-B lead to increased hyperplasia of the endometrial epithelium and inflammation, and no decidualization in the endometrial stroma [93]. Taken together these data indicate that PR-A is critical for implantation, and that PR-B is involved in endometrial differentiation. Female mice knockout for both PR-A and PR-B are infertile, showing severely reduced or absent ovulation, uterine hyperplasia, absence of decidualization, severely limited mammary gland development, and an impaired sexual behavior [94]. After progesterone binding, PR activates a series of signal transductions, involving both genomic and non-genomic pathways. The non-genomic response is rapid and based on the interaction with c-Src kinase to induce the pro-proliferative ERK/MAPK and AKT pathways, important for peri-implantation stromal proliferation [90,95,96]. The genomic action of PR involves its translocation into the nucleus and modulation of gene expression [90]. In the uterine epithelium, progesterone promotes the expression of Indian hedgehog (IHH), which in turn induces up-regulation of stromal chicken ovalbumin upstream promoter-transcription factor II (COUPTFII) that regulates stromal bone morphogenetic protein 2 (BMP2) and consequently the decidualization response of the stromal compartment [97,98,99,100]. Progesterone-mediated induction of IHH is also responsible for the down-regulation of MUC1 [98]. As for the effect of progesterone on decidualization, it has been demonstrated that progesterone stimulation induces heart and neural crest derivatives expressed 2 (HAND2), a transcription factor, whose down-regulation in mouse and human fibroblast cells is associated to reduction of decidualization markers [101].

The genomic response to progesterone action also regulates the expression of transcription factors of the homeobox family [102]. Homeobox protein-A10 and -A11 (HOXA10, HOXA11), are expressed in stromal and glandular compartments of the endometrium throughout the menstrual cycle and are both essential for pregnancy, since their deletion in mice results in implantation defects [103,104,105]. Both HOXA10 and HOXA11 have a role in decidualization [104,106]. HOXA10 positively regulates the expression of the decidual marker Insulin Growth Factor Binding Protein 1 (IGFBP1) [107], while HOXA11 normally function as a repressor of the decidual marker prolactin (PRL) gene, however in cooperation with FOXO1A it induces its 3-fold increase [104]. In vitro models have greatly contributed in understanding the role of progesterone in decidualization [108,109]. Treatment of endometrial organ cultures or endometrial derived stromal cells with progesterone induces expression of PRL [109,110], but with higher efficiency if steroid hormones are used in combination with cAMP [111,112]. cAMP alone can induce decidualization of human endometrial stromal cells (HESCs) but for few days only [113,114,115], since for the stabilization of the process it is necessary the presence of both cAMP and progesterone [112].

In addition to the steroid hormones produced by the ovary, other hormones are involved in the establishment of pregnancy, among which one of the most studied is the chorionic gonadotropin (CG). CG is produced by the trophectoderm of the blastocyst and is one of the main players involved in endometrium–embryo crosstalk at the time of implantation. The ovaries respond to CG, which acts as an agonist of LH, by maintaining the corpus luteum, thus producing the progesterone necessary for the establishment and progression of pregnancy [116]. The responses of the endometrium are multiple, including the inhibition of apoptosis, which usually occurs at the end of the menstrual cycle, by activating anti-apoptotic genes as B-cell lymphoma 2 (BCL-2) [117,118], and the induction of the decidualization process [118,119,120]. Both epithelial and stromal cells possess the LH/CG receptor (LHCGR), a seven transmembrane G protein-coupled receptor, which shows the highest expression during the secretory phase of the menstrual cycle [119,121,122]. Endometrial epithelial cells respond to CG by expressing cyclooxygenase-2 (COX2) and prostaglandin E synthase (PGES), through the activation of extracellular signal-regulated protein kinases 1/2 (Erk1/2) signaling pathway. The increased production of prostaglandin E2 (PGE2) [122,123,124] induces cAMP in endometrial stromal cells and promotes their decidualization [124,125]. COX-derived PGE2 plays an important role in the increase of endometrial vascular permeability, which characterizes the inflammatory reaction typical of implantation [126,127]. In endometrial stromal cells CG activates Erk1/2 signaling pathway, thus increasing the expression of the PR and regulating the expression of genes controlling endometrial receptivity [121]. Moreover, in primates, endometrial stromal cells respond to CG and progesterone by activating Notch receptor 1 (NOTCH1) pathway, as discussed later. NOTCH1 induces the expression of α-smooth muscle actin (α-SMA), which positively regulates remodeling of cytoskeleton and the initial changes typical of the decidualization process [128]. Subsequently, a decrease in CG and NOTCH1 levels is necessary for the completion of decidualization, which is accompanied by an increase in the expression of insulin-like growth factor binding protein-1 (IGFBP1) and prolactin (PRL), markers of decidualization [129,130,131], and a downregulation of LHCGR [120,132,133,134].

### 2.4. Role of Pinopodes

One of the major structural changes of the endometrium during the luteal phase is the formation of apical protrusions on the epithelial cells called pinopodes (also known as uterodomes). These dome-like structures are formed in response to progesterone, but regress upon estrogen stimulation [135,136,137,138]. The function of pinopodes is not clear. Some authors suggest that pinopodes are responsible of pinocytosis and endocytosis of uterine fluid and macromolecules, which facilitates adhesion of the blastocyst to the endometrium [139,140]; others have suggested that they might be directly involved in blastocyst–endometrial interaction through the expression of adhesion molecules, such as integrins [141,142,143,144], or of LIF [145], although co-localization of these molecules and pinopodes has been questioned [146,147].

Pinopodes formation has been initially demonstrated to coincide with the WOI [137], hence their role as potential markers of endometrial receptivity was proposed [148,149]. However, this role is still currently a topic of great debate. Several studies demonstrated that pinopode are present beyond the WOI [150], questioning their utility to identify endometrial receptivity. Moreover, no major differences in the coverage and morphology of pinopodes was observed in endometrial samples from fertile women compared to those of women with recurrent pregnancy loss, suggesting no direct correlation between pinopode density/morphology and pregnancy success [151]. However, recent studies re-evaluated pinopode utility to identify endometrial receptivity, by demonstrating a strong correlation between pinopode quality and pregnancy rate [152,153,154]. These contrasting results may be explained, at least in part, by sampling variability, and lack of standardization for morphological identification and staging of the pinopodes. As recently reported, computer-assisted analysis of endometrial tissue images could be used to overcome operator subjectivity [140]. It should also be considered that absence or presence of pinopodes might not be the solely parameter to consider for endometrial receptivity, as quality and molecular content of pinopodes could also be of relevance.

### 2.5. Growth Factor of the EGF Family

Uterine receptivity is also regulated by members of the epidermal growth factor (EGF) family, whose expression pattern in the peri-implantation uterus has been widely investigated in murine models [155,156,157,158,159,160,161,162]. Among the EGF family members, amphiregulin (AREG) has been identified in the luminal epithelium exclusively at the site of blastocyst apposition and its expression appears to correlate initially with the increase of progesterone levels and then with the attachment reaction [157]. Similarly, the expression of heparin binding-EGF (HB-EGF), which is under the control of both estrogen and progesterone [160], requires the presence of a competent blastocyst and it occurs in the luminal epithelium when pinopodes are fully formed at the site of blastocyst apposition [155,161], while epiregulin (EREG) is expressed in both the luminal epithelium and stroma during blastocyst attachment [158]. This unique expression pattern suggests a role for AREG, HB-EGF, and EREG in uterine receptivity and subsequent embryo adhesion. The role of HB-EGF in blastocyst adhesion to the uterus has been further demonstrated in vitro in a co-culture of a mouse cell line synthesizing transmembrane human HB-EGF (TM HB-EGF) and mouse blastocysts. Cells synthesizing TM HB-EGF adhered to mouse blastocysts more than parental cells or cells synthesizing a constitutively secreted form of HB-EGF [163]. These results are confirmed by a more recent study using HB-EGF mutant mice, which demonstrates that maternal deficiency of HB-EGF limits pregnancy success [162].

### 2.6. NOTCH Signaling Pathway

NOTCH signaling pathway is involved in the regulation of various cellular processes such as cell proliferation, invasion, adhesion, survival, apoptosis and differentiation [164,165,166,167]. All four NOTCH receptors, the ligands Jagged1 (JAG1) and Delta-like (DLL) 4 and the target genes hairy enhancer of split (HES) and Hes-related 1 (HEY1) are known to be expressed by the endometrium [168,169,170,171]. Several ligands and receptors of the NOTCH signaling pathway are expressed in both the inner cell mass (ICM) and trophectoderm of the human blastocyst [172,173,174]. NOTCH1 plays an important role in the process of decidualization, by inducing pro-survival signals in the endometrium, thus avoiding apoptosis normally occurring at the end of the menstrual cycle. Hess et al. showed that blastocyst-conditioned medium induces an increase in the expression of NOTCH family members in decidual cells, suggesting a role for this pathway in decidualization [175]. Moreover, it has recently been shown that NOTCH signaling pathway is dysregulated in the endometrium of women with unexplained recurrent pregnancy loss [176]. Activation of NOTCH1 pathway in the endometrium is stimulated by CG and progesterone and leads to increased expression of α-SMA and Forkhead box protein O1 (FOXO1) [1,128,177]. FOXO1, in turns, induces expression of PRL and IGFBP1 and it is essential for the decidualization process [178,179,180,181,182]. NOTCH1 is involved in the inhibition of cAMP/protein kinase A (PKA) signaling pathway [183], so that NOTCH1 has to be downregulated to allow cAMP response of stromal cells. Similar to what described for α-SMA and LHCGR expression, a downregulation of NOTCH1 is necessary for the induction of IGFBP1 and the completion of decidualization [111,120,128].

### 2.7. Interleukin-1b in Blastocyst–Endometrium Dialogue

Interleukin (IL)-1β is another important factor supporting blastocyst–endometrium dialogue, playing a fundamental role in decidualization of stromal cells and in successful blastocyst implantation. IL-1β is secreted by cytotrophoblast cells isolated from first trimester placenta, while its expression is lower in cultures from second and third trimester placenta [184]. In endometrial stromal cells IL-1β induces the expression of COX2 and PGE2, known to increase the levels of cAMP, which are necessary for decidualization, as above described [185,186]. Moreover, in vivo infusion of IL-1β and CG promotes the expression of IGFBP1 in apical surface stromal cells [133]. It has been demonstrated that inhibition of COX2 in human and baboon endometrial stromal cells is able to block the decidualization induced by IL-1β in the presence of steroid hormones, suggesting that IL-1β acts upstream of COX2 [185]. On the contrary, inhibition of COX2 does not affect decidualization induced by cAMP and steroid hormones, suggesting that cAMP acts downstream of COX2 and PGE2 [185]. Interestingly, cAMP is able to block decidualization induced by IL-1β indicating a negative feedback between IL-1β and cAMP [185,187]. In baboon, IL-1β positively regulates the expression of matrix metalloproteinase (MMP) 3 in endometrial stroma, thus inducing degradation of the ECM. Considering that disruption of the ECM might reflect in cellular cytoskeleton remodeling, IL-1β may play an important role in the decidualization also by promoting cytoskeleton changes typical of this process [188,189]. All these data clearly indicate that IL-1β plays a relevant role in blastocyst–endometrium crosstalk.

### 2.8. Thyroid Hormone in Endometrial Receptivity

Endometrial receptivity is regulated also by thyroid hormone (TH). Both thyroid hormone and thyroid-stimulating hormone receptors (TR and TSHR, respectively) are expressed in the endometrium with variations during the menstrual cycle [190]. Two of the isoforms of TR, TRα1, and TRβ1, are expressed during the mid-luteal phase in glandular and luminal epithelium, showing an increase during the secretory phase, followed by a drastic decrease. Interestingly, the expression of TRα1 and TRβ1, and also of TRα2 and TSHR, in endometrial cells is concomitant to the appearance of the pinopodes and the establishment of endometrial receptivity. The expression of TRα1, TRβ1, TRα2 and also of type 2 deiodinase (DIO2) is regulated by progesterone. In fact, the administration of mifepristrone, an anti-progestinic drug that makes the endometrium unreceptive and induces menstrual bleeding, reduces the expression of TRα1 and TRα2, while it up-regulates TRβ1 and DIO2 expression, suggesting a role for progesterone in regulating molecules involved in TH synthesis and metabolism [191]. The role of TH pathway in endometrial function is also demonstrated by the observation that hypothyroidism is able to reduce uterine endometrial thickness, and also interferes with estrogenic response of the endometrium [192]. TH regulates endometrial receptivity also by acting on LIF pathway, since TSH induces increased expression of LIF and LIF receptor (LIFR) in endometrial stromal cells obtained from human endometrial samples, suggesting a major role for TSH in the implantation process [190].

### 2.9. Immune Cells in Implantation

A role for the immune system in embryo implantation has been widely investigated for obvious reasons. The decidua plays a fundamental role in ensuring immune tolerance toward the semi-allogenic conceptus, protecting it from the mother’s immune system. Regulatory T cells (Tregs) are CD4+ CD25+ T cells, having the role to suppress the immune response [193]. During early pregnancy, in the decidua there is an increase in Tregs, which produce immunosuppressive cytokines, such as IL-10, for inducing immune tolerance [194,195,196,197]. Other cells involved in maternal immune tolerance are the uNK, a particular type of NK cells, which lose their cytotoxic functions during pregnancy andplay a supportive role by enhancing angiogenesis. uNK cells induce immune tolerance by reducing inflammation through interferon-γ (IFN-γ) [198] and by inhibiting the function of T cells through the expression of immunomodulatory molecules such as galectin-1 and glycodelin A [199].

### 2.10. Endometrial Receptivity Array

Recently, a customized endometrial receptivity array (ERA), containing 238 genes related to endometrial receptivity, was created [200]. These genes, differentially expressed in the receptive phase, encode for factors involved in several biological processes, such as processes related to the immune system, circulation, response to external stimulus, behavior, cell cycle, cell adhesion, anatomical structure development, cell–cell signaling, and mitotic cell cycle [200]. Beside the many above mentioned genes suggested to regulate endometrial receptivity, additional genes have been identified by ERA, highlighting the great complexity of factors regulating implantation. ERA has been suggested as a more accurate and reproducible approach to assess endometrial receptivity compared to histological analysis [201] and its use has been proposed for RIF patients [202]. Considering how critical the molecular signature of the endometrium is for embryo implantation, a test which unequivocally assess if the embryo and the uterus are in synchrony may be of great value to avoid ineffective embryo transfers. However, the utility of ERA in the clinical practice is still debated [203]. More recently, a smaller set of genes has been proposed to assess the receptivity status of the endometrium in biopsies obtained in the secretory phase [204]. It is reasonable to foresee new additional advances in this area, that is of potential great clinical utility in the management of infertile women undergoing IVF, as well as in women with RIF and unexplained RPL.

## 3. Implantation of the Competent Blastocyst

In order for a healthy pregnancy to proceed, the embryo needs to synchronize its developmental program with endometrial receptivity and to acquire the ability to implant, defined as competence. A competent blastocyst is characterized by distinctive morphological and molecular features, which are discussed in this section.

### 3.1. Transport, orientation and hatching

#### 3.1.1. Blastocyst Transport and Orientation

After fertilization, the embryo encased in a non-anchored glycocalix, the so-called zona pellucida, which prevents ectopic implantation, descends the Fallopian tube and reaches the uterine cavity, while undergoing profound morphological changes ending in the formation of the blastocyst [205,206]. For successful implantation into the maternal tissues, a correct orientation of the blastocyst towards the uterine wall is needed. In most eutherian mammals, at the time of first contact of the blastocyst with the endometrial epithelium, the ICM of the various embryos has an almost constantly specific orientation toward the uterus. In humans, the ICM faces the uterine wall. This positioning of the ICM usually correlates with the site of trophectoderm attachment to the endometrium, as well as with subsequent development of the fetal membranes and placental structures [207,208]. In rodents, implantation occurs in anti-mesometrial position with the ICM facing the mesometrium [209]. Why, within most species, the ICM of the blastocyst, or the placenta, should be positioned consistently in the same way with respect to the uterine wall is not completely understood. Moreover, how the blastocyst becomes correctly oriented [210,211] or what directs the process has not been well clarified, for even the most commonly-studied mammals. However, it has been postulated that orientation depends on signals from the endometrium rather than from the embryo, since embryo-mimicking structures (beads, bubbles or cells) end up in the position that the embryo would occupy [212,213,214,215]. For example, in mice endometrial expression of the transcriptional regulator Rbpj is required to instruct embryo orientation, and its conditional deletion determines loss of ventral-dorsal orientation [216]. A role for endometrial glands in embryo orientation has been also proposed. Indeed, recent data indicate that in mice endometrial gland development is confined to the anti-mesometrial side of the uterus [217]. In consideration of the above discussed essential role of uterine glands in implantation, it can be speculated that glands may drive the anti-mesometrial orientation of the implanting mouse embryo, possibly through the expression of specific factors. In this respect, it has been shown that Wnt signaling activity in the mouse uterus is limited to the anti-mesometrial region and a role for Wnt proteins in anti-mesometrial localization of the implanting embryo has been proposed [217].

#### 3.1.2. Blastocyst Hatching

**Figure 1:**
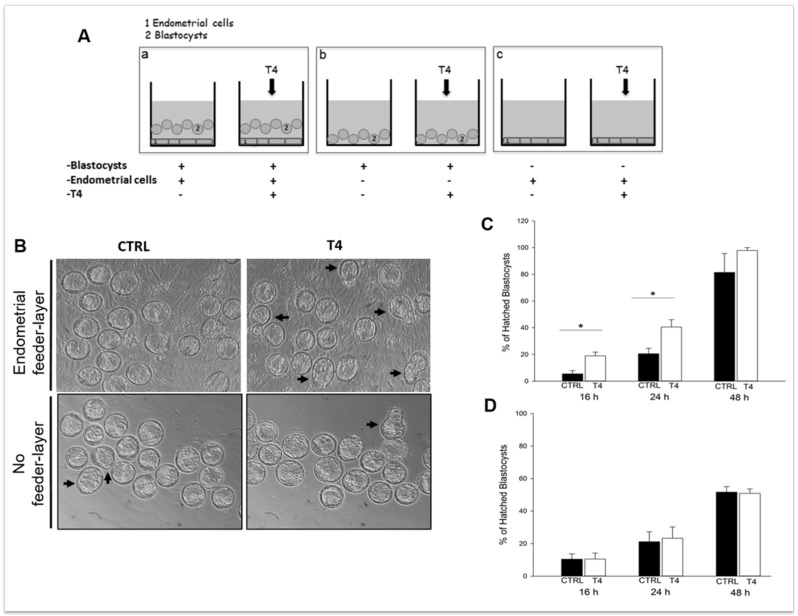
Thyroid hormone (TH) supplementation stimulates mouse blastocyst hatching in vitro. (**A**) Schematic representation of the in vitro model developed to assess TH role in implantation. (**a**) Co-culture of murine blastocysts and endometrial primary cells as the feeder layer; (**b**) blastocysts cultured on plastic; and (**c**) endometrial cells cultured without blastocysts. (**B**) Representative images of the cultures. Scale bar 50 µm. (**C**,**D**) Graphs summarizing the results shown in B: percent of hatched blastocysts after co-culture on endometrial cells (**C**) or on plastic (**D**). Reproduced with permission from Piccirilli et al. [223].

Embedding of the blastocyst into the maternal endometrium requires hatching from the zona pellucida, which otherwise would prevent adhesion of the embryo to the uterine wall. Blastocyst hatching exposes the trophectoderm and allows the blastocyst to implant in the maternal uterus. The crucial event for blastocyst hatching is the formation of a nick into the zona pellucida, and proteases, such as serine-, cysteine-, and metallo-proteases have been proposed to play a major role in this event depending on the species [218,219,220,221,222,223]. Cathepsins, belonging to the ubiquitous cysteine proteases family [224], have been demonstrated to be involved in blastocyst hatching and zona lysis in mice; the expression of cathepsin L and P (mRNA and protein) and their natural inhibitor, Cystatin C, has been demonstrated in mouse peri-hatching blastocysts [225]; treatment of golden hamster embryos with Cystatin C is able to block blastocyst hatching [221]. The process of murine blastocyst hatching from the zona pellucida is also regulated by two mouse-specific proteinases, Strypsin (ISP1) and Lysin (ISP2). ISP1 and ISP2 are two related S1-family serine proteinases, which are tandemly localized in a cluster of tryptase genes [226,227]. The ISPs are co-expressed in the mouse preimplantation embryos and in the mouse uterine endometrium during the WOI, indicating that they could play a role in the process of blastocyst implantation [226,228]. Expression of ISP genes is positively regulated by progesterone and TH [219,223,226] and ISPs are secreted by the blastocyst and the endometrial glands into uterine fluid just prior to implantation [229]. The use of antibodies against ISP1/ISP2 abrogate murine embryo hatching and outgrowth, ascribing a crucial role for ISPs in this process [228]. This is further supported by our recent observations using mouse blastocysts cultured in the presence of TH, with or without endometrial cells used as the feeder layer. In the presence of endometrial feeder cells, TH is able to anticipate blastocyst hatching (Figure 1) by upregulating the expression of blastocyst produced ISPs, and to enhance blastocyst outgrowth by upregulating endometrial ISPs and MMPs. On the contrary, in the absence of the endometrial feeder layer, TH does not affect blastocyst hatching, suggesting that TH is one of the players involved in the bidirectional crosstalk between the blastocyst and the endometrium during the WOI [223]. Human homologs of ISPs have not been so far identified, and it is possible that other proteases might be involved in blastocyst hatching in humans.

### 3.2. Apposition

**Figure 2:**
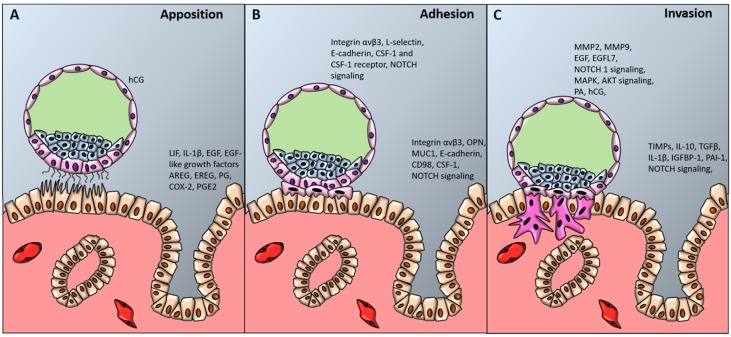
Blastocyst apposition, adhesion and invasion. The diagram shows a preimplantation-stage (**A**, **B**) and invading (**C**) blastocyst (about 9 to 10 days after conception) and the processes and factors required for uterine receptivity and blastocyst apposition (**A)**, adhesion (**B**) and invasion (**C**). hCG denotes human chorionic gonadotropin, LIF leukemia inhibiting factor, IL-1β interleukin-1 beta, EGF-like growth factors epidermal growth factor-like growth factors, AREG amphiregulin, EREG epiregulin, PG progesterone, COX-2 cyclooxygenase-2, PGE2 prostaglandin E2, CSF-1 colony stimulating factor-1, NOTCH1 Notch receptor 1, OPN osteopontin, MUC-1 mucin-1, MMPs metalloproteinases, EGFL7 epidermal growth factor-like domain 7, MAPK mitogen activated protein kinase, AKT protein kinase B, PA plasminogen activator, TGFβ transforming growth factor beta, TIMPs tissue inhibitor of metalloproteinases, PAI-1 plasminogen activator inhibitor-1.

Histological analysis of uteri of pregnant women allows to recognize three different levels of blastocyst adhesion to the uterine wall, which correspond to the three stages of blastocyst implantation (Figure 2) [230,231].

#### 3.2.1. LIF Signaling

Blastocyst apposition is the initial stage representing the first physical contact between the blastocyst and the endometrium, in which the blastocyst finds a site for implantation, guided by the maternal endometrium [232,233]. The site of implantation in the human uterus is usually in the upper and posterior part in the midsagittal plane. During blastocyst apposition, the microvilli placed on the apical surface of trophectoderm interdigitate with the pinopodes localized on the apical surface of the uterine epithelium (Figure 2A). These specialized structures support a stable binding between trophoblast and uterine epithelial cells, so that the plasma membranes of these cells are parallel and separated by a distance of 20 nm [234]. The pinopodes secrete LIF [145]. LIF is a cytokine of the IL-6 family, which in the uterus activates the Janus kinases (JAK)-signal transducer and activator of transcription protein (STAT) pathway, and therefore phosphorylates STAT3, whose activation is required for implantation [235,236]. LIF is indispensable for blastocyst implantation. Mice knockout for LIF are infertile. Although able to develop blastocysts, these mice show implantation failure; however successful implantation occurs in surrogate mothers [90]. In Lif-null mice the expression of EGF-like growth factors, such as HB-EGF, AREG, and EREG, which, as previously mentioned, are normally expressed by the luminal epithelium adjacent to the blastocyst and are essential for successful pregnancy, is abolished [237]. Since the defects in decidualization caused by the absence of LIF can be rescued by intrauterine administration of EGF ligand [238], it has been hypothesized that LIF favors blastocyst invasion by reducing the expression of cell–cell junction molecules and proliferation of the stromal cells through activation of EGF signaling pathway [239]. In fertile women, LIF expression increases in the endometrium around the time of implantation, while infertile women express low levels of this factor [240,241].

#### 3.2.2. Chorionic Gonadotropin

Once a competent blastocyst makes contact with the maternal endometrium, a dialogue made of signals and responses between them occurs. One of the most important factors secreted by trophoblast cells is CG. CG is expressed very early by the embryo, since its mRNA can be detected starting from the 6–8 cell stage. The protein is secreted by both zona enclosed or hatched blastocysts, and is independent of blastocyst interaction with the endometrium [242]. During pregnancy, CG is firstly detectable in maternal blood during implantation and then rapidly increases [243]. As discussed before, CG plays a fundamental role in inducing the production of progesterone and mediating the decidualization process, thus allowing implantation of the blastocyst.

### 3.3. Adhesion

#### 3.3.1. Adhesion Molecules Mediating Blastocyst Adhesion

Following apposition, stable adhesion of the blastocyst to the endometrium occurs, mediated by the interaction between several receptors and ligands (Figure 2B). Over the last decades, several of these ligands and receptors have been identified. It has been observed that both the pinopodes of the endometrial epithelium and the trophectoderm of the blastocyst express the integrin αvβ3, together with the endometrial expression of its ligand, the glycoprotein osteopontin (OPN). Their expression during the WOI suggests a role in implantation [160,244,245], and the binding between integrin αvβ3 and its ligand OPN might mediate the stable adhesion between the trophoblast and the endometrium [246]. Using an in vitro model of implantation, Genbacev et al. suggested that trophoblast adhesion to the uterine wall is also mediated by L-selectin expressed on the surface of the trophoblast cells, and uterine epithelial oligosaccharide ligands, such as HECA-452 and MECA-79 [247,248]. More recently it has been also demonstrated that the transmembrane glycoprotein MUC1, abundantly expressed at the apical surface of uterine epithelium under the control of progesterone, acts as a scaffold mediating the binding between L-selectin and their ligands [249].

The adhesion of the blastocyst to the endometrium is also promoted by the expression of adhesion molecules, such as cadherins. The presence of endothelial cadherin (E-cadherin) in both the trophoblasts and endometrial epithelium, regulated by progesterone, indicates that it may play an important role in blastocyst adhesion to the endometrium [250]. As trophoblast cells proliferate, differentiate and invade the stroma, they downregulate E-cadherin and increase osteoblast cadherin (OB-cadherin) [251,252]. This temporal expression of OB-cadherin in the endometrial epithelium suggests that this adhesion molecule later mediates trophoblast–endometrium interactions. Blastocyst adhesion is also favored by the expression of the glycoproteic receptor CD98 on the surface of endometrial cells, which is normally involved not only in amino acids transport but also in cell fusion [253,254]. Using two human endometrial cell lines characterized by low and high receptivity, Dominguez et al. demonstrated that CD98 receptor is significantly associated with the receptive phenotype. In human endometrial samples, CD98 expression was spatially restricted to the apical surface of endometrial cells and temporally restricted to the WOI. Treatment of primary endometrial epithelial cells with hCG, 17-β-estradiol, LIF, or EGF increases expression of CD98, greatly enhancing murine blastocyst adhesion, while its siRNA-mediated depletion reduced blastocyst adhesion rate [255].

#### 3.3.2. NOTCH Signaling in Blastocyst Adhesion to the Endometrium

The expression of NOTCH receptors and ligands in the trophectoderm of the blastocyst and that of NOTCH1, DLL4, and JAG1 in the apical surface of the endometrial epithelium during the mid-secretory phase [170,256] would suggest a role for NOTCH signaling in the adhesion of the blastocyst to the epithelium. Indeed, it has been demonstrated that blastocyst-conditioned medium regulates NOTCH1 and JAG1 expression in the endometrial epithelium [256], suggesting that the blastocyst is able to activate NOTCH signaling in the endometrium, thus possibly regulating its receptivity. This is reinforced by the fact that women with primary infertility show a reduced or absent immunostaining for JAG1 in the luminal epithelium during the mid-secretory phase [256].

#### 3.3.3. Colony-Stimulating Factor-1 in Implantation

A role for colony-stimulating factor-1 (CSF-1) in implantation has been proposed. Indeed, supplementation of CSF-1 in cultures of human trophoblast cells promotes their differentiation in syncytiotrophoblast cells and leads to the production of placental lactogen [257]. In addition, supplementation of CSF-1 to cultures of murine blastocyst induces trophoblast outgrowth [258]. However, using osteopetrotic mutant mice, which lack CSF-1, it has been shown that a maternal source of CSF-1 is not necessary for pregnancy, and possibly the fetus can provide a source of CSF-1 which compensate for the absence of maternally produced CSF-1 [259].

### 3.4. Invasion

Finally, in the third stage, invasion occurs, starting with the penetration of highly invasive trophoblast cells in the uterine epithelium (Figure 2C), followed by infiltration in the basement membrane and in the stromal compartment, a process known as “interstitial invasion” [233,260,261]. Besides invading the endometrial stroma, trophoblast cells also migrate down the lumen of maternal spiral arteries, replace the vascular endothelial lining and become embedded in the arterial walls. This process of “endovascular invasion” allows to replace small-caliber, high-resistance vessels with large-caliber, low-resistance vessels, ensuring an adequate blood supply to the fetoplacental unit [262,263]. Defects in trophoblast endovascular invasion of maternal spiral arteries can seriously impair placental function, leading to significant complications of advanced gestation, such as intrauterine growth restriction (IUGR) and preeclampsia [264].

#### 3.4.1. Matrix Metalloproteinases in Blastocyst Invasion

The huge invasive ability of the fetal trophoblast is due to a high production of activated gelatinases, in particular MMPs 2 and 9 [265,266,267]. Trophoblastic MMPs are regulated in response to IL-1β, tumor necrosis factor alpha (TNFα) IL-1α, macrophage colony-stimulating factor (MCSF), transforming growth factor β (TGFβ), IGFBP1, leptin, hCG, and EGF [124,268,269,270,271,272], which are secreted from different cell types at the feto-maternal interface, such as trophoblasts themselves and endometrial cells, promoting trophoblast invasion. As already mentioned above, the expression of MMPs involved in endometrial invasion by trophoblast cells is also under the control of TH, as TH positively regulates MMP expression by endometrial cells [223].

#### 3.4.2. Epidermal Growth Factor-Like Domain 7

Recently, we demonstrated that migration and invasion of trophoblast cells is regulated by the secreted factor Epidermal growth factor-like domain 7 (EGFL7), a novel NOTCH interactor. EGFL7 activates NOTCH1, MAPK and AKT signaling pathways in both trophoblast cell lines and primary cells [273]. Activation of the NOTCH pathway is important in both interstitial and endovascular invasion by trophoblast cells. In vitro functional assays show that invasion of Matrigel by trophoblast cells overexpressing EGFL7 is impaired in the presence of a γ-secretase inhibitor, normally used to inhibit NOTCH activation [264,273]. NOTCH appears to be also involved in trophoblast endovascular invasion, since uNK, involved in the disruption of endometrial spiral arteries integrity, express NOTCH1 and 2 and maternal cells surrounding spiral arteries express DLL1 [264], and NOTCH activation may lead to arterial wall disruption. These results are further confirmed by the fact that NOTCH pathway is dysregulated in placenta of women affected by preeclampsia [264,274,275,276,277,278,279,280], a common pregnancy disorder characterized by an insufficient trophoblast invasion and an inadequate vascular remodeling. In women affected by preeclampsia, the alteration of NOTCH pathway is accompanied by a concomitant altered expression of EGFL7, in both placenta and maternal circulation [274,281].

#### 3.4.3. Endometrial Control of Blastocyst Invasion

In all the placental species the extent of endometrial decidualization is proportional to the invasiveness of the embryo. The human placenta is the most invasive one known so far, and it has been suggested that the unique invasiveness of the human trophoblast could be due to its high production of hyperglycosylated CG isoform, which is maximal in the first weeks of pregnancy [282,283].

In order to limit the extent of trophoblast invasion, both trophoblast and endometrium balance the expression of growth factors, cytokines, and enzymes. As an example, maternal endometrium increases the production of tissue inhibitors of MMPs (TIMPs), due to a spatial and temporal regulation of cytokines and growth factors, such as IL-10 [284], TGFβ and IL-1α [268]. While IL-1α significantly increases the activity of MMP-9 and MCSF increases MMP-9 immunoreactivity, TGFβ inhibits total gelatinolytic activity, MMP-9 activity and immunoreactivity [268]. TIMP-3, which is up-regulated by progesterone, plays a major role in restricting trophoblast invasion by limiting ECM degradation, and its expression has been detected in the fetal extravillous trophoblast, as well as in the maternal endometrial cells [285,286]. On the contrary, by in situ hybridization in implanting mouse embryos no expression was observed for TIMP-1 or TIMP-2 in the embryo proper, trophoblasts, or in the decidua. Weak signals were demonstrated for TIMP-1 only in the circular layer of myometrial smooth muscle and in some uterine stroma cells distant from the site of embryo implantation. Moreover, the expression of TIMP-1 and TIMP-2 is not dependent on the stage of the menstrual cycle [286]. Trophoblast invasion is promoted by the action of the plasminogen activator (PA) system since it is able to promote trophoblast invasion, by converting plasminogen into the active serine protease plasmin, which in turn, degrades ECM [287]. In endometrial cells, TGFβ regulates trophoblast invasion up-regulating the expression of plasminogen activator inhibitor-1 (PAI-1), which is the main inhibitor of urokinase-type plasminogen activator (uPA) [288,289,290], and decorin, a decidua-derived TGFβ binding proteoglycan, which inhibits proliferation, migration and invasion of trophoblast cells [291]. The blastocyst is completely embedded in the uterine stroma 8 days after fertilization and the site of entry is covered by fibrin, over which the uterine epithelial cells grow [233,292,293].

#### 3.4.4. Blastocyst Competence Profiling in ARTs

Although many of the molecular players involved in the complex process of implantation have been characterized, the selection of competent embryos remains one of the major challenges in ART. A parallel and complementary morphological and molecular profiling analysis of the embryo may represent a successful approach for embryo selection, thus improving IVF outcome. Although morphological characteristics have been significantly associated with euploidy and competence of the embryo [294,295], their evaluation for good quality embryo selection has some limitations, such as operator subjectivity, variability linked to the timing of laboratory observation, culture medium and other culture conditions, hence combined different approaches might be useful [296]. In this respect, metabolomic and proteomic analyses of embryo spent media have been proposed as complementary, non-invasive tools to select embryos with higher implantation ability [297,298,299]. Limitations derive from the variability of commercial culture media, high metabolic plasticity of the embryos which can adapt to different culture conditions and from the fact that embryo development and metabolism vary under different culture conditions [300]. Recently, novel strategies based on gene expression profiling of trophectoderm biopsies have been developed and have linked gene expression patterns with developmental competence [301,302,303]. Although complementary approaches may be used to select the best embryos to be transferred, it should be considered that it has been recently proposed that it is the endometrium itself that selectively discriminates between high-and low-quality embryos in order to guarantee a successful implantation. Based on this, it could be envisioned a test in which the endometrium might be used as a “bio-sensor” to avoid transfer of low-quality embryos, which if implanted would be possibly later rejected resulting in a miscarriage [304,305].

## 4. Conclusions

Over the last decades the research aimed to reveal the biomolecular processes and pathways underlying animal and human implantation has greatly progressed for two major reasons. On one hand, the exciting advances in available technologies allowed to define in depth the factors and the pathways involved in proper implantation. On the other hand, the introduction of ARTs and their spectacular development in response to the increasing clinical demands from infertile patients allowed to better understand the determinants of a successful implantation and of several conditions of reproductive failure. Additionally, the large diffusion of ARTs provided new perspectives for studies on implantation, making available biological samples previously unavailable for research; follicular fluid, granulosa cells, oocytes, embryos, culture medium of embryos, and blastocysts are examples of this.

As a general concept, it has become clear that reproduction in humans can be considered a rather inefficient process and in several ways is different from reproduction in other species. An emerging concept is that the proper molecular crosstalk between endometrium and blastocyst is of paramount relevance to ensure a proper implantation. In this context, the studies on animal models, apart for the above differences, may greatly help to increase current knowledge. The specific roles of blastocyst and endometrium are being discovered, although much progress still has to be done in this field. The final objective of this field of research effort is twofold: (1) to improve the understanding of how reproduction and implantation evolved and differentiated among the species; (2) to offer more and more effective treatment options to patients with infertility, RIF and RPL.

## Abbreviations

WOI	Window of implantation
CG	Chorionic gonadotropin
IL	Interleukin
LIF IVF RIF	Leukemia inhibitory factor In vitro fertilization Recurrent implantation failure
ART	Assisted reproduction technology
ECM	Extracellular matrix
LH uNK	Luteinizing hormone Uterine natural killer
cAMP	Cyclic adenosine monophosphate
BCL-2 LHCGR	B-cell lymphoma 2 LH/CG receptor
COX2	Cyclooxygenase-2
PGES	Prostaglandin E synthase
Erk1/2	Extracellular signal-regulated protein kinases 1/2
PGE2 NOTCH1	Prostaglandin E2 Notch receptor 1
PR	Progesterone receptor
α-SMA	α-smooth muscle actin
IGFBP1	Insulin-like growth factor binding protein-1
ER	Oestrogen receptor
PRL	Prolactin
EGF	Epidermal growth factor
AREG	Amphiregulin
HB-EGF	Heparin binding epidermal growth factor
EREG	Epiregulin
JAG1	Jagged1
DLL	Delta-like
HES	Hairy enhancer of split
HEY1	Hes-related 1
ICM	Inner cell mass
FOXO1	Forkhead box protein O1
PKA	Protein kinase A
MMP	Matrix metalloproteinase
TH	Thyroid hormone
TR	Thyroid hormone receptor
TSHR	Thyroid-stimulating hormone receptor
DIO2	Type 2 deiodinase
LIFR	LIF receptor
ERA	Endometrial receptivity array
HESCs	Human endometrial stromal cells
Tregs	Regulatory T cells
IFN-γ	Interferon-γ
ISP1	Strypsin
ISP2	Lysin
JAK	Janus kinases
STAT	Signal transducer and activator of transcription protein
OPN	Osteopontin
MUC1	Mucin 1
E-cadherin	Endothelial cadherin
OB-cadherin	Osteoblast cadherin
CSF-1	Colony-stimulating factor-1
IUGR	Intrauterine growth restriction
TNFα	Tumor necrosis factor α
MCSF	Macrophage colony-stimulating factor
TGFβ	Transforming growth factor β
EGFL7	Epidermal growth factor-like domain 7
TIMPs	Tissue inhibitors of MMPs
PA	Plasminogen activator
PAI-1	Plasminogen activator inhibitor-1
uPA	Urokinase-type plasminogen activator

## References

[1] Su R.W., Fazleabas A.T. (2015). Implantation and Establishment of Pregnancy in Human and Nonhuman Primates. Adv. Anat. Embryol. Cell Biol..

[2] Fazleabas A.T., Strakova Z. (2002). Endometrial function: Cell specific changes in the uterine environment. Mol. Cell. Endocrinol..

[3] Tabibzadeh S., Babaknia A. (1995). The signals and molecular pathways involved in implantation, a symbiotic interaction between blastocyst and endometrium involving adhesion and tissue invasion. Hum. Reprod..

[4] Norwitz E.R., Schust D.J., Fisher S.J. (2001). Implantation and the survival of early pregnancy. N. Engl. J. Med..

[5] Sharkey A.M., Smith S.K. (2003). The endometrium as a cause of implantation failure. Best Pract. Res. Clin. Obstet. Gynaecol..

[6] Paria B.C., Huet-Hudson Y.M., Dey S.K. (1993). Blastocyst’s state of activity determines the “window” of implantation in the receptive mouse uterus. Proc. Natl. Acad. Sci. USA.

[7] Psychoyos A., Greep R.O., Astwood E.G., Geiger S.R. (1973). Endocrine control of egg implantation. Handbook of Physiology.

[8] Kim S.M., Kim J.S. (2017). A Review of Mechanisms of Implantation. Dev. Reprod..

[9] Wilcox A.J., Weinberg C.R., O’Connor J.F., Baird D.D., Schlatterer J.P., Canfield R.E., Armstrong E.G., Nisula B.C. (1988). Incidence of early loss of pregnancy. N. Engl. J. Med..

[10] World Health Organization Multiple Definitions of Infertility. https://www.who.int/reproductivehealth/topics/infertility/multiple-definitions/en/.

[11] Gurunath S., Pandian Z., Richard A.R., Bhattacharya S. (2011). Defining infertility a systematic review of prevalence studies. Hum. Reprod. Update.

[12] Boivin J., Bunting L., Collins J., Nygren K. (2007). International estimates of infertility prevalence and treatmentseeking: Potential need and demand for infertility medical care. Hum. Reprod..

[13] Mascarenhas M.N., Flaxman S.R., Boerma T., Vanderpoel S., Stevens G.A. (2012). National, regional, and global trends in infertility prevalence since 1990: A systematic analysis of 277 health surveys. PLoS Med..

[14] European Society of Human Reproduction and Embryology. https://www.eshre.eu/Press-Room/Resources.

[15] National Health Service Causes of Infertility. https://www.nhs.uk/conditions/infertility/.

[16] Abrao M.S., Muzii L., Marana R. (2013). Anatomical causes of female infertility and their management. Int. J. Gynaecol. Obstet..

[17] Unuane D., Tournaye H., Velkeniers B., Poppe K. (2011). Endocrine disorders & female infertility. Best Pract. Res. Clin. Endocrinol. Metab..

[18] Mekinian A., Cohen J., Alijotas-Reig J., Carbillon L., Nicaise-Roland P., Kayem G., Daraï E., Fain O., Bornes M. (2016). Unexplained Recurrent Miscarriage and Recurrent Implantation Failure: Is There a Place for Immunomodulation?. Am. J. Reprod. Immunol..

[19] Ticconi C., Pietropolli A., Di Simone N., Piccione E., Fazleabas A. (2019). Endometrial Immune Dysfunction in Recurrent Pregnancy Loss. Int. J. Mol. Sci..

[20] Larsen E.C., Christiansen O.B., Kolte A.M., Macklon N. (2013). New insights into mechanisms behind miscarriage. BMC Med..

[21] Bashiri A., Halper K.I., Orvieto R. (2018). Recurrent Implantation Failure-update overview on etiology, diagnosis, treatment and future directions. Reprod. Biol. Endocrinol..

[22] Coughlan C., Ledger W., Wang Q., Liu F., Demirol A., Gurgan T., Cutting R., Ong K., Sallam H., Li T.C. (2014). Recurrent implantation failure: Definition and management. Reprod. Biomed. Online.

[23] Practice Committee of the American Society for Reproductive Medicine (2012). Evaluation and treatment of recurrent pregnancy loss: A committee opinion. Fertil. Steril..

[24] ESHRE Early Pregnancy Guideline Development Group (2017). Recurrent Pregnancy Loss.

[25] Psychoyos A. (1986). Uterine receptivity for nidation. Ann. N.Y. Acad. Sci..

[26] Psychoyos A., Lizuka R., Semm K. (1988). The ‘implantation window’: Can it be enlarged or displaced?. Human Reproduction. Current Status/Future Prospect.

[27] Ma W.G., Song H., Das S.K., Paria B.C., Dey S.K. (2003). Estrogen is a critical determinant that specifies the duration of the window of uterine receptivity for implantation. Proc. Natl. Acad. Sci. USA.

[28] Blesa D., Ruiz-Alonso M., Simon C. (2014). Clinical management of endometrial receptivity. Semin. Reprod. Med..

[29] Donaghay M., Lessey B.A. (2007). Uterine receptivity: Alterations associated with benign gynecological disease. Semin. Reprod. Med..

[30] Navot D., Scott R.T., Droesch K., Veeck L.L., Liu H.C., Rosenwaks Z. (1991). The window of embryo transfer and the efficiency of human conception in vitro. Fertil. Steril..

[31] Franchi A., Zaret J., Zhang X., Bocca S., Oehinger S. (2008). Expression of immunomodulatory genes, their protein products and specific ligands/receptors during the window of implantation in the human endometrium. Mol. Hum. Reprod..

[32] Altmäe S., Reimand J., Hovatta O., Zhang P., Kere J., Laisk T., Saare M., Peters M., Vilo J., Stavreus-Evers A. (2012). Research resource: Interactome of human embryo implantation: Identification of gene expression pathways, regulation, and integrated regulatory networks. Mol. Endocrinol..

[33] Koot Y.E., Macklon N.S. (2013). Embryo implantation: Biology, evaluation, and enhancement. Curr. Opin. Obstet. Gynecol..

[34] Lessey B.A. (2011). Assessment of endometrial receptivity. Fertil. Steril..

[35] Bartol F.F., Wiley A.A., Floyd J.G., Ott T.L., Bazer F.W., Gray C.A., Spencer T.E. (1999). Uterine differentiation as a foundation for subsequent fertility. J. Reprod. Fertil. Suppl..

[36] Gray C.A., Bartol F.F., Tarleton B.J., Wiley A.A., Johnson G.A., Bazer F.W., Spencer T.E. (2001). Developmental biology of uterine glands. Biol. Reprod..

[37] Spencer T.E., Hayashi K., Hu J., Carpenter K.D. (2005). Comparative developmental biology of the mammalian uterus. Curr. Top. Dev. Biol..

[38] Cooke P.S., Ekman G.C., Kaur J., Davila J., Bagchi I.C., Clark S.G., Dziuk P.J., Hayashi K., Bartol F.F. (2012). Brief exposure to progesterone during a critical neonatal window prevents uterine gland formation in mice. Biol. Reprod..

[39] Hayashi K., Yoshioka S., Reardon S.N., Rucker E.B., Spencer T.E., Demayo F.J., Lydon J.P., Maclean J.A. (2011). WNTs in the neonatal mouse uterus: Potential regulation of endometrial gland development. Biol. Reprod..

[40] Miller C., Sassoon D.A. (1998). Wnt-7a maintains appropriate uterine patterning during the development of the mouse female reproductive tract. Development.

[41] Mericskay M., Kitajewski J., Sassoon D. (2004). Wnt5a is required for proper epithelial-mesenchymal interactions in the uterus. Development.

[42] Dunlap K.A., Filant J., Hayashi K., Rucker E.B., Song G., Deng J.M., Behringer R.R., DeMayo F.J., Lydon J., Jeong J.W. (2011). Postnatal deletion of Wnt7a inhibits uterine gland morphogenesis and compromises adult fertility in mice. Biol. Reprod..

[43] Franco H.L., Dai D., Lee K.Y., Rubel C.A., Roop D., Boerboom D., Jeong J.W., Lydon J.P., Bagchi I.C., Bagchi M.K. (2011). WNT4 is a key regulator of normal postnatal uterine development and progesterone signaling during embryo implantation and decidualization in the mouse. FASEB J..

[44] Jeong J.W., Lee H.S., Franco H.L., Broaddus R.R., Taketo M.M., Tsay S.Y., Lydon J.P., DeMayo F.J. (2009). Beta-catenin mediates glandular formation and dysregulation of beta-catenin induces hyperplasia formation in the murine uterus. Oncogene.

[45] Farah O., Biechele S., Rossant J., Dufort D. (2018). Regulation of porcupine-dependent Wnt signaling is essential for uterine development and function. Reproduction.

[46] Reardon S.N., King M.L., MacLean J.A., Mann J.L., DeMayo F.J., Lydon J.P., Hayashi K. (2012). CDH1 is essential for endometrial differentiation, gland development, and adult function in the mouse uterus. Biol. Reprod..

[47] Guimarães-Young A., Neff T., Dupuy A.J., Goodheart M.J. (2016). Conditional deletion of Sox17 reveals complex effects on uterine adenogenesis and function. Dev. Biol..

[48] Cheong Y., Boomsa C., Heijnen C., Macklon N. (2013). Uterine secretomics: A window on the maternal-embryo interface. Fertil. Steril..

[49] Salamonsen L.A., Edgell T., Rombauts L.J., Stephens A.N., Robertson D.M., Rainczuk A., Nie G., Hannan N.J. (2013). Proteomics of the human endometrium and uterine fluid: A pathway to biomarker discovery. Fertil. Steril..

[50] Hempstock J., Cindrova-Davies T., Jauniaux E., Burton G.J. (2004). Endometrial glands as a source of nutrients, growth factors and cytokines during the first trimester of human pregnancy: A morphological and immunohistochemical study. Reprod. Biol. Endocrinol..

[51] Kane M.T., Morgan P.M., Coonan C. (1997). Peptide growth factors and preimplantation development. Hum. Reprod..

[52] Hannan N.J., Stephens A.N., Rainczuk A., Hincks C., Rombauts L.J., Salamonsen L.A. (2010). 2D-DiGE analysis of the human endometrial secretome reveals differences between receptive and nonreceptive states in fertile and infertile women. J. Proteome Res..

[53] Salamonsen L.A., Hannan N.J., Dimitriadis E. (2007). Cytokines and chemokines during human embryo implantation: Roles in implantation and early placentation. Semin. Reprod. Med..

[54] Vilella F., Ramirez L.B., Simon C. (2013). Lipidomics as an emerging tool to predict endometrial receptivity. Fertil. Steril..

[55] Burton G.J., Scioscia M., Rademacher T.W. (2011). Endometrial secretions: Creating a stimulatory microenvironment within the human early placenta and implications for the aetiopathogenesis of preeclampsia. J. Reprod. Immunol..

[56] Burton G.J., Jauniaux E., Charnock-Jones D.S. (2007). Human early placental development: Potential roles of the endometrial glands. Placenta.

[57] Guzeloglu-Kayisli O., Kayisli U.A., Taylor H.S. (2009). The role of growth factors and cytokines during implantation: Endocrine and paracrine interactions. Semin. Reprod. Med..

[58] Boomsma C.M., Kavelaars A., Eijkemans M.J., Lentjes E.G., Fauser B.C., Heijnen C.J., Macklon N.S. (2009). Endometrial secretion analysis identifies a cytokine profile predictive of pregnancy in IVF. Hum. Reprod..

[59] Hannan N.J., Paiva P., Meehan K.L., Rombauts L.J., Gardner D.K., Salamonsen L.A. (2011). Analysis of fertility-related soluble mediators in human uterine fluid identifies VEGF as a key regulator of embryo implantation. Endocrinology.

[60] Heng S., Hannan N.J., Rombauts L.J., Salamonsen L.A., Nie G. (2011). PC6 levels in uterine lavage are closely associated with uterine receptivity and significantly lower I a subgroup of women with unexplained infertility. Hum. Reprod..

[61] Zhang Y., Wang Q., Wang H., Duan E. (2017). Uterine Fluid in Pregnancy: A Biological and Clinical Outlook. Trends Mol. Med..

[62] Ladines-Llave C.A., Maruo T., Manalo A.S., Mochizuki M. (1991). Cytologic localization of epidermal growth factor and its receptor in developing human placenta varies over the course of pregnancy. Am. J. Obstet. Gynecol..

[63] Mühlhauser J., Crescimanno C., Kaufmann P., Höfler H., Zaccheo D., Castellucci M. (1993). Differentiation and proliferation patterns in human trophoblast revealed by c-erbB-2 oncogene product and EGF-R. J. Histochem. Cytochem..

[64] Sharkey A.M., King A., Clark D.E., Burrows T.D., Johki P.P., Charnock Jones D.S., Loke Y.W., Smith S.K. (1999). Localization of leukaemia inhibitory factor and its receptor in human placenta thoughout pregnancy. Biol. Reprod..

[65] Kojima K., Kanzaki H., Iwai M., Hatayama H., Fujimoto M., Narukawa S., Higuchi T., Kaneko Y., Mori T., Fujita T. (1995). Expression of leukaemia inhibitory factor (LIR) receptor in human placenta: A possible role for LIF in the growth and differentiation of trophoblasts. Hum. Reprod..

[66] Cooper J.C., Sharkey A.M., McLaren J., Charnock Jones D.S., Smith S.K. (1995). Localization of vascular endothelial growth factor and its receptor, flt, in human placenta and decidua by immunohistochemistry. J. Reprod. Fertil..

[67] Wewer U.M., Faber M., Liotta L.A., Albrechtsen R. (1985). Immunochemical and ultrastructural assessment of the nature of the pericellular basement membrane of human decidual cells. Lab. Investig..

[68] Wynn R.M. (1974). Ultrastructural development of the human decidua. Am. J. Obstet. Gynecol..

[69] Dunn C.L., Kelly R.W., Critchley H.O. (2003). Decidualization of the human endometrial stromal cell: An enigmatic transformation. Reprod. Biomed. Online.

[70] Kim J.J., Jaffe R.C., Fazleabas A.T. (1999). Blastocyst invasion and the stromal response in primates. Hum. Reprod..

[71] Ramathal C.Y., Bagchi I.C., Taylor R.N., Bagchi M.K. (2010). Endometrial decidualization: Of mice and men. Semin. Reprod. Med..

[72] Teklenburg G., Salker M., Molokhia M., Lavery S., Trew G., Aojanepong T., Mardon H.J., Lokugamage A.U., Rai R., Landles C. (2010). Natural selection of human embryos: Decidualizing endometrial stromal cells serve as sensors of embryo quality upon implantation. PLoS ONE.

[73] Macklon N.S., Brosens J.J. (2014). The human endometrium as a sensor of embryo quality. Biol. Reprod..

[74] Chen M., Wolfe A., Wang X., Chang C., Yeh S., Radovick S. (2009). Generation and characterization of a complete null estrogen receptor alpha mouse using Cre/LoxP technology. Mol. Cell. Biochem..

[75] Lee H.R., Kim T.H., Choi K.C. (2012). Functions and physiological roles of two types of estrogen receptors, ERα and ERβ, identified by estrogen receptor knockout mouse. Lab. Anim. Res..

[76] Lubahn D.B., Moyer J.S., Smithies O., Golding T.S., Couse J.F., Korach K.S. (1993). Alteration of reproductive function but not prenatal sexual development after insertional disruption of the mouse estrogen receptor gene. Proc. Natl. Acad. Sci. USA.

[77] Hapangama D.K., Kamal A.M., Bulmer J.N. (2015). Estrogen receptor β: The guardian of the endometrium. Hum. Reprod. Update.

[78] Cha J., Sun X., Dey S.K. (2012). Mechanisms of implantation: Strategies for successful pregnancy. Nat. Med..

[79] Thomas K., De Hertogh R., Pizarro M., Van Exter C., Ferin J. (1973). Plasma LH-HCG, 17-estradiol, estrone and progesterone monitoring around ovulation and subsequent nidation. Int. J. Fertil..

[80] Stefkovich M.L., Arao Y., Hamilton K.J., Korach K.S. (2018). Experimental models for evaluating non-genomic estrogen signaling. Steroids.

[81] Lee C.H., Kim T.H., Lee J.H., Oh S.J., Yoo J.Y., Kwon H.S., Kim Y.I., Ferguson S.D., Ahn J.Y., Ku B.J. (2013). Extracellular signal-regulated kinase 1/2 signaling pathway is required for endometrial decidualization in mice and human. PLoS ONE.

[82] Zhu L., Pollard J.W. (2007). Estradiol-17beta regulates mouse uterine epithelial cell proliferation through insulin-like growth factor 1 signaling. Proc. Natl. Acad. Sci. USA.

[83] Klotz D.M., Hewitt S.C., Ciana P., Raviscioni M., Lindzey J.K., Foley J., Maggi A., DiAugustine R.P., Korach K.S. (2002). Requirement of estrogen receptor-alpha in insulin-like growth factor-1 (IGF-1)-induced uterine responses and in vivo evidence for IGF-1/estrogen receptor cross-talk. J. Biol. Chem..

[84] Hewitt S.C., Lierz S.L., Garcia M., Hamilton K.J., Gruzdev A., Grimm S.A., Lydon J.P., DeMayo F.J., Korach K.S. (2019). A distal super enhancer mediates estrogen-dependent mouse uterine-specific gene transcription of Insulin-like growth factor 1 (Igf1). J. Biol. Chem..

[85] Tsai S.J., Wu M.H., Chen H.M., Chuang P.C., Wing L.Y. (2002). Fibroblast growth factor-9 is an endometrial stromal growth factor. Endocrinology.

[86] Wang W., Li Q., Bagchi I.C., Bagchi M.K. (2010). The CCAAT/enhancer binding protein beta is a critical regulator of steroid-induced mitotic expansion of uterine stromal cells during decidualization. Endocrinology.

[87] Surveyor G.A., Gendler S.J., Pemberton L., Das S.K., Chakraborty I., Julian J., Pimental R.A., Wegner C.C., Dey S.K., Carson D.D. (1995). Expression and steroid hormonal control of Muc-1 in the mouse uterus. Endocrinology.

[88] Rosario G.X., Stewart C.L. (2016). The Multifaceted Actions of Leukaemia Inhibitory Factor in Mediating Uterine Receptivity and Embryo Implantation. Am. J. Reprod. Immunol..

[89] Stewart C.L., Kaspar P., Brunet L.J., Bhatt H., Gadi I., Kontgen F., Abbondanzo S.J. (1992). Blastocyst implantation depends on maternal expression of leukaemia inhibitory factor. Nature.

[90] Patel B., Elguero S., Thakore S., Dahoud W., Bedaiwy M., Mesiano S. (2015). Role of nuclear progesterone receptor isoforms in uterine pathophysiology. Hum. Reprod. Update.

[91] Paulson R.J. (2011). Hormonal induction of endometrial receptivity. Fertil. Steril..

[92] Mulac-Jericevic B., Lydon J.P., DeMayo F.J., Conneely O.M. (2003). Defective mammary gland morphogenesis in mice lacking the progesterone receptor B isoform. Proc. Natl. Acad. Sci. USA.

[93] Mulac-Jericevic B., Mullinax R.A., DeMayo F.J., Lydon J.P., Conneely O.M. (2000). Subgroup of reproductive functions of progesterone mediated by progesterone receptor-B isoform. Science.

[94] Lydon J.P., DeMayo F.J., Funk C.R., Mani S.K., Hughes A.R., Montgomery C.A., Shyamala G., Conneely O.M., O’Malley B.W. (1995). Mice lacking progesterone receptor exhibit pleiotropic reproductive abnormalities. Genes Dev..

[95] Boonyaratanakornkit V., Scott M.P., Ribon V., Sherman L., Anderson S.M., Maller J.L., Miller W.T., Edwards D.P. (2001). Progesterone receptor contains a proline-rich motif that directly interacts with SH3 domains and activates c-Src family tyrosine kinases. Mol. Cell.

[96] Vallejo G., La Greca A.D., Tarifa-Reischle I.C., Mestre-Citrinovitz A.C., Ballare C., Beato M., Saragueta P. (2014). CDC2 mediates progestin initiated endometrial stromal cell proliferation: A PR signaling to gene expression independently of its binding to chromatin. PLoS ONE.

[97] Lee D.K., Kurihara I., Jeong J.W., Lydon J.P., DeMayo F.J., Tsai M.J., Tsai S.Y. (2010). Suppression of ERalpha activity by COUP-TFII is essential for successful implantation and decidualization. Mol. Endocrinol..

[98] Takamoto N., Zhao B., Tsai S.Y., DeMayo F.J. (2002). Identification of Indian hedgehog as a progesterone-responsive gene in the murine uterus. Mol. Endocrinol..

[99] Lee K., Jeong J., Kwak I., Yu C.T., Lanske B., Soegiarto D.W., Toftgard R., Tsai M.J., Tsai S., Lydon J.P. (2006). Indian hedgehog is a major mediator of progesterone signaling in the mouse uterus. Nat. Genet..

[100] Kurihara I., Lee D.K., Petit F.G., Jeong J., Lee K., Lydon J.P., DeMayo F.J., Tsai M.J., Tsai S.Y. (2007). COUP-TFII mediates progesterone regulation of uterine implantation by controlling ER activity. PLoS Genet..

[101] Huyen D.V., Bany B.M. (2011). Evidence for a conserved function of heart and neural crest derivatives expressed transcript 2 in mouse and human decidualization. Reproduction.

[102] Du H., Taylor H.S. (2015). The Role of Hox Genes in Female Reproductive Tract Development, Adult Function, and Fertility. Cold Spring Harb. Perspect. Med..

[103] Benson G.V., Lim H., Paria B.C., Satokata I., Dey S.K., Maas R.L. (1996). Mechanisms of reduced fertility in Hoxa-10 mutant mice: Uterine homeosis and loss of maternal Hoxa-10 expression. Development.

[104] Lim H., Ma L., Ma W.G., Maas R.L., Dey S.K. (1999). Hoxa-10 regulates uterine stromal cell responsiveness to progesterone during implantation and decidualization in the mouse. Mol. Endocrinol..

[105] Gendron R.L., Paradis H., Hsieh-Li H.M., Lee D.W., Potter S.S., Markoff E. (1997). Abnormal uterine stromal and glandular function associated with maternal reproductive defects in Hoxa-11 null mice. Biol. Reprod..

[106] Lynch V.J., Brayer K., Gellersen B., Wagner G.P. (2009). HoxA-11 and FOXO1A cooperate to regulate decidual prolactin expression: Towards inferring the core transcriptional regulators of decidual genes. PLoS ONE.

[107] Kim J.J., Taylor H.S., Akbas G.E., Foucher I., Trembleau A., Foucher I., Trembleau A., Jaffe R.C., Fazleabas A.T., Unterman T.G. (2003). Regulation of insulin-like growth factor binding protein-1 promoter activity by FKHR and HOXA10 in primate endometrial cells. Biol. Reprod..

[108] Maslar I.A., Ansbacher R. (1986). Effects of progesterone on decidual prolactin production by organ cultures of human endometrium. Endocrinology.

[109] Daly D.C., Maslar I.A., Riddick D.H. (1983). Prolactin production during in vitro decidualization of proliferative endometrium. Am. J. Obstet. Gynecol..

[110] Tabanelli S., Tang B., Gurpide E. (1992). In vitro decidualization of human endometrial stromal cells. J. Steroid Biochem. Mol. Biol..

[111] Kim J.J., Jaffe R.C., Fazleabas A.T. (1998). Comparative studies on the in vitro decidualization process in the baboon (Papio anubis) and human. Biol. Reprod..

[112] Brosens J.J., Hayashi N., White J.O. (1999). Progesterone receptor regulates decidual prolactin expression in differentiating human endometrial stromal cells. Endocrinology.

[113] Telgmann R., Maronde E., Taskén K., Gellersen B. (1997). Activated protein kinase A is required for differentiation-dependent transcription of the decidual prolactin gene in human endometrial stromal cells. Endocrinology.

[114] Samalecos A., Reimann K., Wittmann S., Schulte H.M., Brosens J.J., Bamberger A.M., Gellersen B. (2009). Characterization of a novel telomerase-immortalized human endometrial stromal cell line, St-T1b. Reprod. Biol. Endocrinol..

[115] Popovici R.M., Kao L.C., Giudice L.C. (2000). Discovery of new inducible genes in in vitro decidualized human endometrial stromal cells using microarraytechnology. Endocrinology.

[116] Hirose T. (1920). Exogenous stimulation of corpus luteum formation in the rabbit: Influence of extracts of human placenta, decidua, fetus, hydatid mole, and corpus luteum on the rabbit gonad. J. Jpn. Gynecol. Soc..

[117] Lovely L.P., Fazleabas A.T., Fritz M.A., McAdams D.G., Lessey B.A. (2005). Prevention of endometrial apoptosis: Randomized prospective comparison of human chorionic gonadotropin versus progesterone treatment in the luteal phase. J. Clin. Endocrinol. Metab..

[118] Jasinska A., Strakova Z., Szmidt M., Fazleabas A.T. (2006). Human chorionic gonadotropin and decidualization in vitro inhibits cytochalasin-D-induced apoptosis in cultured endometrial stromal fibroblasts. Endocrinology.

[119] Reshef E., Lei Z.M., Rao C.V., Pridham D.D., Chegini N., Luborsky J.L. (1990). The presence of gonadotropin receptors in nonpregnant human uterus, human placenta, fetal membranes, and decidua. J. Clin. Endocrinol. Metab..

[120] Cameo P., Szmidt M., Strakova Z., Mavrogianis P., Sharpe-Timms K.L., Fazleabas A.T. (2006). Decidualization regulates the expression of the endometrial chorionic gonadotropin receptor in the primate. Biol. Reprod..

[121] Tapia-Pizarro A., Archiles S., Argandoña F., Valencia C., Zavaleta K., Cecilia Johnson M., González-Ramos R., Devoto L. (2017). hCG activates Epac-Erk1/2 signaling regulating Progesterone Receptor expression and function in human endometrial stromal cells. Mol. Hum. Reprod..

[122] Banerjee P., Sapru K., Strakova Z., Fazleabas A.T. (2009). Chorionic gonadotropin regulates prostaglandin E synthase via a phosphatidylinositol 3-kinase-extracellular regulatory kinase pathway in a human endometrial epithelial cell line: Implications for endometrial responses for embryo implantation. Endocrinology.

[123] Zhou X.L., Lei Z.M., Rao C.V. (1999). Treatment of human endometrial gland epithelial cells with chorionic gonadotropin/luteinizing hormone increases the expression of the cyclooxygenase-2 gene. J. Clin. Endocrinol. Metab..

[124] Srisuparp S., Strakova Z., Brudney A., Mukherjee S., Reierstad S., Hunzicker-Dunn M., Fazleabas A.T. (2003). Signal transduction pathways activated by chorionic gonadotropin in the primate endometrial epithelial cells. Biol. Reprod..

[125] Tanaka N., Miyazaki K., Tashiro H., Mizutani H., Okamura H. (1993). Changes in adenylyl cyclase activity in human endometrium during the menstrual cycle and in human decidua during pregnancy. J. Reprod. Fertil..

[126] Van der Weiden R.M., Helmerhorst F.M., Keirse M.J. (1991). Influence of prostaglandins and platelet activating factor on implantation. Hum. Reprod..

[127] Lim H., Paria B.C., Das S.K., Dinchuk J.E., Langenbach R., Trzaskos J.M., Dey S.K. (1997). Multiple female reproductive failures in cyclooxygenase 2-deficient mice. Cell.

[128] Afshar Y., Miele L., Fazleabas A.T. (2012). Notch1 is regulated by chorionic gonadotropin and progesterone in endometrial stromal cells and modulates decidualization in primates. Endocrinology.

[129] Christian M., Pohnke Y., Kempf R., Gellersen B., Brosens J.J. (2002). Functional association of PR and CCAAT/enhancer-binding protein beta isoforms: Promoter-dependent cooperation between PR-B and liverenriched inhibitory protein, or liver-enriched activatory protein and PR-A in human endometrial stromal cells. Mol. Endocrinol..

[130] Gao J., Mazella J., Tang M., Tseng L. (2000). Ligand-activated progesterone receptor isoform hPR-A is a stronger transactivator than hPR-B for the expression of IGFBP-1 (insulin-like growth factor binding protein-1) in human endometrial stromal cells. Mol. Endocrinol..

[131] Gellersen B., Brosens I.A., Brosens J.J. (2007). Decidualization of the human endometrium: Mechanisms, functions, and clinical perspectives. Semin. Reprod. Med..

[132] Christensen S., Verhage H.G., Nowak G., de Lanerolle P., Fleming S., Bell S.C., Fazleabas A.T., Hild-Petito S. (1995). Smooth muscle myosin II and alpha smooth muscle actin expression in the baboon (Papio anubis) uterus is associated with glandular secretory activity and stromal cell transformation. Biol. Reprod..

[133] Strakova Z., Mavrogianis P., Meng X., Hastings J.M., Jackson K.S., Cameo P., Brudney A., Knight O., Fazleabas A.T. (2005). In vivo infusion of interleukin-1beta and chorionic gonadotropin induces endometrial changes that mimic early pregnancy events in the baboon. Endocrinology.

[134] Tarantino S., Verhage H.G., Fazleabas A.T. (1992). Regulation of insulin-like growth factor-binding proteins in the baboon (Papio anubis) uterus during early pregnancy. Endocrinology.

[135] Develioglu O.H., Hsiu J.G., Nikas G., Toner J.P., Oehninger S., Jones H.W. (1999). Endometrial estrogen and progesterone receptor and pinopode expression in stimulated cycles of oocyte donors. Fertil. Steril..

[136] Stavreus-Evers A., Nikas G., Sahlin L., Eriksson H., Landgren B.M. (2001). Formation of pinopodes in human endometrium is associated with the concentrations of progesterone and progesterone receptors. Fertil. Steril..

[137] Martel D., Monier M.N., Roche D., Psychoyos A. (1991). Hormonal dependence of pinopode formation at the uterine luminal surface. Hum. Reprod..

[138] Singh M.M., Trivedi R.N., Chauhan S.C., Srivastava V.M., Makker A., Chowdhury S.R., Kamboj V.P. (1996). Uterine estradiol and progesterone receptor concentration, activities of certain antioxidant enzymes and dehydrogenases and histoarchitecture in relation to time of secretion of nidatory estrogen and high endometrial sensitivity in rat. J. Steroid Biochem. Mol. Biol..

[139] Parr M.B., Parr E.L. (1974). Uterine luminal epithelium: Protrusions mediate endocytosis, not apocrine secretion, in the rat. Biol. Reprod..

[140] Matson B.C., Pierce S.L., Espenschied S.T., Holle E., Sweatt I.H., Davis E.S., Tarran R., Young S.L., Kohout T.A., van Duin M. (2017). Adrenomedullin improves fertility and promotes pinopodes and cell junctions in the peri-implantatio endometrium. Biol. Reprod..

[141] Lessey B.A. (2003). Two pathways of progesterone action in the human endometrium: Implications for implantation and contraception. Steroids.

[142] Peyghambari F., Salehnia M., Forouzandeh Moghadam M., Rezazadeh Valujerdi M., Hajizadeh E. (2010). The correlation between the endometrial integrins and osteopontin expression with pinopodes development in ovariectomized mice in response to exogenous steroids hormones. Iran. Biomed. J..

[143] Liu S., Hua T., Xin X., Shi R., Chi S., Wang H. (2017). Altered expression of hormone receptor, integrin β3 and pinopode in the endometrium of luteal phase defect women. Gynecol. Endocrinol..

[144] Qian Z.-D., Weng Y., Wang C.F., Huang L.L., Zhu X.M. (2017). Research on the expression of integrin β3 and leukaemia inhibitory factor in the decidua of women with cesarean scar pregnancy. BMC Pregnancy Childbirth.

[145] Kabir-Salmani M., Nikzad H., Shiokawa S., Akimoto Y., Iwashita M. (2005). Secretory role for human pinopodes (pinopods): Secretion of LIF. Mol. Hum. Reprod..

[146] Mikołajczyk M., Skrzypczak J., Wirstlein P. (2011). No correlation between pinopod formation and LIF and MMP2 expression in endometrium during implantation window. Folia Histochem. Cytobiol..

[147] Creus M., Ordi J., Fábregues F., Casamitjana R., Ferrer B., Coll E., Vanrell J.A., Balasch J. (2002). alphavbeta3 integrin expression and pinopod formation in normal and out-of-phase endometria of fertile and infertile women. Hum. Reprod..

[148] Nikas G., Aghajanova L. (2002). Endometrial pinopodes: Some more understanding on human implantation?. Reprod. Biomed..

[149] Lopata A., Bentin-Ley U., Enders A. (2002). “Pinopodes” and implantation. Rev. Endocr. Metab. Disord..

[150] Quinn C., Ryan E., Claessens E.A., Greenblatt E., Hawrylyshyn P., Cruickshank B., Hannam T., Dunk C., Casper R.F. (2007). The presence of pinopodes in the human endometrium does not delineate the implantation window. Fertil. Steril..

[151] Xu B., Sun X., Li L., Wu L., Zhang A., Feng Y. (2012). Pinopodes, leukemia inhibitory factor, integrin-beta3, and mucin-1 expression in the peri-implantation endometrium of women with unexplained recurrent pregnancy loss. Fertil. Steril..

[152] Qiong Z., Jie H., Yonggang W., Bin X., Jing Z., Yanping L. (2017). Clinical validation of pinopode as a marker of endometrial receptivity: A randomized controlled trial. Fertil. Steril..

[153] Jin X.Y., Zhao L.J., Luo D.H., Liu L., Dai Y.D., Hu X.X., Wang Y.Y., Lin X., Hong F., Li T.C. (2017). Pinopode score around the time of implantation is predictive of successful implantation following frozen embryo transfer in hormone replacement cycles. Hum. Reprod..

[154] Aunapuu M., Kibur P., Jarveots T., Arend A. (2018). Changes in Morphology and Presence of Pinopodes in Endometrial Cells during the Luteal Phase in Women with Infertility Problems: A Pilot Study. Medicina.

[155] Das S.K., Wang X.N., Paria B.C., Damm D., Abraham J.A., Klagsbrun M., Andrews G.K., Dey S.K. (1994). Heparin-binding EGF-like growth factor gene is induced in the mouse uterus temporally by the blastocyst solely at the site of its apposition: A possible ligand for interaction with blastocyst EGF-receptor in implantation. Development.

[156] Lim H., Dey S.K., Das S.K. (1997). Differential expression of the erbB2 gene in the periimplantation mouse uterus: Potential mediator of signaling by epidermal growth factor-like growth factors. Endocrinology.

[157] Das S.K., Chakraborty I., Paria B.C., Wang X.N., Plowman G., Dey S.K. (1995). Amphiregulin is an implantation-specific and progesterone-regulated gene in the mouse uterus. Mol. Endocrinol..

[158] Das S.K., Das N., Wang J., Lim H., Schryver B., Plowman G.D., Dey S.K. (1997). Expression of beta cellulin and epiregulin genes in the mouse uterus temporally by the blastocyst solely at the site of its apposition is coincident with the “window” of implantation. Dev. Biol..

[159] Lim H., Das S.K., Dey S.K. (1998). erbB genes in the mouse uterus: Cell-specific signaling by epidermal growth factor (EGF) family of growth factors during implantation. Dev. Biol..

[160] Lessey B.A. (2002). Adhesion molecules and implantation. J. Reprod. Immunol..

[161] Stavreus-Evers A., Aghajanova L., Brismar H., Eriksson H., Landgren B.M., Hovatta O. (2002). Co-existence of heparin-binding epidermal growth factor-like growth factor and pinopodes in human endometrium at the time of implantation. Mol. Hum. Reprod..

[162] Xie H., Wang H., Tranguch S., Iwamoto R., Mekada E., Demayo F.J., Lydon J.P., Das S.K., Dey S.K. (2007). Maternal heparin-binding-EGF deficiency limits pregnancy success in mice. Proc. Natl. Acad. Sci. USA.

[163] Raab G., Kover K., Paria B.C., Dey S.K., Ezzell R.M., Klagsbrun M. (1996). Mouse preimplantation blastocysts adhere to cells expressing the transmembrane form of heparin-binding EGF-like growth factor. Development.

[164] Artavanis-Tsakonas S., Rand M.D., Lake R.J. (1999). Notch signaling: Cell fate control and signal integration in development. Science.

[165] Bray S.J. (2006). Notch signaling: A simple pathway becomes complex. Mol. Cell Biol..

[166] Leong K.G., Karsan A. (2006). Recent insights into the role of Notch signaling in tumorigenesis. Blood.

[167] Rizzo P., Miao H., D’Souza G., Osipo C., Song L.L., Yun J., Zhao H., Mascarenhas J., Wyatt D., Antico G. (2008). Cross-talk between notch and the estrogen receptor in breast cancer suggests novel therapeutic approaches. Cancer. Res..

[168] Cobellis L., Caprio F., Trabucco E., Mastrogiacomo A., Coppola G., Manente L., Colacurci N., De Falco M., De Luca A. (2008). The pattern of expression of Notch protein members in normal and pathological endometrium. J. Anat..

[169] Mitsuhashi Y., Horiuchi A., Miyamoto T., Kashima H., Suzuki A., Shiozawa T. (2012). Prognostic significance of Notch signaling molecules and their involvement in the invasiveness of endometrial carcinoma cells. Histopathology.

[170] Mazella J., Liang S., Tseng L. (2008). Expression of Delta-like protein 4 in the human endometrium. Endocrinology.

[171] Mikhailik A., Mazella J., Liang S., Tseng L. (2009). Notch ligand-dependent gene expression in human endometrial stromal cells. Biochem. Biophys. Res. Commun..

[172] Adjaye J., Huntriss J., Herwig R., BenKahla A., Brink T.C., Wierling C., Hultschig C., Groth D., Yaspo M.L., Picton H.M. (2005). Primary differentiation in the human blastocyst: Comparative molecular portraits of inner cell mass and trophectoderm cells. Stem Cells.

[173] Aghajanova L., Shen S., Rojas A.M., Fisher S.J., Irwin J.C., Giudice L.C. (2012). Comparative transcriptome analysis of human trophectoderm and embryonic stem cell-derived trophoblasts reveal key participants in early implantation. Biol. Reprod..

[174] Wang Q.T., Piotrowska K., Ciemerych M.A., Milenkovic L., Scott M.P., Davis R.W., Zernicka-Goetz M. (2004). A genome-wide study of gene activity reveals developmental signaling pathways in the preimplantation mouse embryo. Dev. Cell.

[175] Hess A.P., Hamilton A.E., Talbi S., Dosiou C., Nyegaard M., Nayak N., Genbecev-Krtolica O., Mavrogianis P., Ferrer K., Kruessel J. (2007). Decidual stromal cell response to paracrine signals from the trophoblast: Amplification of immune and angiogenic modulators. Biol. Reprod..

[176] Strug M.R., Su R.W., Kim T.H., Mauriello A., Ticconi C., Lessey B.A., Young S.L., Lim J.M., Jeong J.W., Fazleabas A.T. (2018). RBPJ mediates uterine repair in the mouse and is reduced in women with recurrent pregnancy loss. FASEB J..

[177] Strug M.R., Su R., Young J.E., Dodds W.G., Shavell V.I., Díaz-Gimeno P., Ruíz-Alonso M., Simón C., Lessey B.A., Leach R.E. (2016). Intrauterine human chorionic gonadotropin infusion in oocyte donors promotes endometrial synchrony and induction of early decidual markers for stromal survival: A randomized clinical trial. Hum. Reprod..

[178] Brar A.K., Handwerger S., Kessler C.A., Aronow B.J. (2001). Gene induction and categorical reprogramming during in vitro human endometrial fibroblast decidualization. Physiol. Genom..

[179] Christian M., Zhang X., Schneider-Merck T., Unterman T.G., Gellersen B., White J.O., Brosens J.J. (2002). Cyclic AMP induced forkhead transcription factor, FKHR, cooperates with CCAAT/enhancer-binding protein beta in differentiating human endometrial stromal cells. J. Biol. Chem..

[180] Buzzio O.L., Lu Z., Miller C.D., Unterman T.G., Kim J.J. (2006). FOXO1A differentially regulates genes of decidualization. Endocrinology.

[181] Grinius L., Kessler C., Schroeder J., Handwerger S. (2006). Forkhead transcription factor FOXO1A is critical for induction of human decidualization. J. Endocrinol..

[182] Labied S., Kajihara T., Madureira P.A., Fusi L., Jones M.C., Higham J.M., Varshochi R., Francis J.M., Zoumpoulidou G., Essafi A. (2006). Progestins regulate the expression and activity of the forkhead transcription factor FOXO1 in differentiating human endometrium. Mol. Endocrinol..

[183] Hallaq R., Volpicelli F., Cuchillo-Ibanez I., Hooper C., Mizuno K., Uwanogho D., Causevic M., Asuni A., To A., Soriano S. (2015). The Notch intracellular domain represses CRE-dependent transcription. Cell. Signal..

[184] Librach C., Feigenbaum S., Bass K., Cui T., Verastas N., Sadovsky Y., Quigley J., French D., Fisher S. (1994). Interleukin-1 beta regulates human cytotrophoblast metalloproteinase activity and invasion in vitro. J. Biol. Chem..

[185] Strakova Z., Srisuparp S., Fazleabas A.T. (2000). Interleukin-1beta induces the expression of insulin-like growth factor binding protein-1 during decidualization in the primate. Endocrinology.

[186] Fazleabas A.T., Kim J.J., Strakova Z. (2004). Implantation: Embryonic signals and the modulation of the uterine environment–a review. Placenta.

[187] Strakova Z., Srisuparp S., Fazleabas A.T. (2002). IL-1beta during in vitro decidualization in primate. J. Reprod. Immunol..

[188] Strakova Z., Szmidt M., Srisuparp S., Fazleabas A.T. (2003). Inhibition of matrix metalloproteinases prevents the synthesis of insulin-like growth factor binding protein-1 during decidualization in the baboon. Endocrinology.

[189] Fazleabas A.T., Bell S.C., Fleming S., Sun J., Lessey B.A. (1997). Distribution of integrins and the extracellular matrix proteins in the baboon endometrium during the menstrual cycle and early pregnancy. Biol. Reprod..

[190] Aghajanova L., Stavreus-Evers A., Lindeberg M., Landgren B.M., Skjoldebrand Sparre L., Hovatta O. (2011). Thyroid-stimulating hormone receptor and thyroid hormone receptors are involved in human endometrial physiology. Fertil. Steril..

[191] Catalano R.D., Critchley H.O., Heikinheimo O., Baird D.T., Hapangama D., Sherwin J.R.A., Charnock-Jones D.S., Smith S.K., Sharkey A.M. (2007). Mifepristone induced progesterone withdrawal reveals novel regulatory pathways in human endometrium. Mol. Hum. Reprod..

[192] Wakim A.N., Polizotto S.L., Buffo M.J., Marrero M.A., Burholt D.R. (1993). Thyroid hormones in human follicular fluid and thyroid hormone receptors in human granulosa cells. Fertil. Steril..

[193] Campbell D.J., Koch M.A. (2011). Phenotypical and functional specialization of FOXP3+ regulatory T cells. Nat. Rev. Immunol..

[194] Tilburgs T., Roelen D.L., van der Mast B.J., de Groot-Swings G.M., Kleijburg C., Scherjon S.A., Claas F.H. (2008). Evidence for a selective migration of fetus-specific CD4 + CD25bright regulatory T cells from the peripheral blood to the decidua in human pregnancy. J. Immunol..

[195] Xiong H., Zhou C., Qi G. (2010). Proportional changes of CD4 + CD25 + Foxp3+ regulatory T cells in maternal peripheral blood during pregnancy and labor at term and preterm. Clin. Investig. Med..

[196] Hara M., Kingsley C.I., Niimi M., Read S., Turvey S.E., Bushell A.R., Morris P.J., Powrie F., Wood K.J. (2001). IL-10 is required for regulatory T cells to mediate tolerance to alloantigens in vivo. J. Immunol..

[197] Robertson S.A., Care A.S., Moldenhauer L.M. (2018). Regulatory T cells in embryo implantation and the immune response to pregnancy. J. Clin. Investig..

[198] Fu B., Li X., Sun R., Tong X., Ling B., Tian Z., Wei H. (2013). Natural killer cells promote immune tolerance by regulating inflammatory TH17 cells at the human maternal-fetal interface. Proc. Natl. Acad. Sci. USA.

[199] Koopman L.A., Kopcow H.D., Rybalov B., Boyson J.E., Orange J.S., Schatz F., Masch R., Lockwood C.J., Schachter A.D., Park P.J. (2003). Human decidual natural killer cells are a unique NK cell subset with immunomodulatory potential. J. Exp. Med..

[200] Díaz-Gimeno P., Horcajadas J.A., Martínez-Conejero J.A., Esteban F.J., Alamá P., Pellicer A., Simón C. (2011). A genomic diagnostic tool for human endometrial receptivity based on the transcriptomic signature. Fertil. Steril..

[201] Díaz-Gimeno P., Ruiz-Alonso M., Blesa D., Bosch N., Martínez-Conejero J.A., Alamá P., Garrido N., Pellicer A., Simón C. (2013). The accuracy and reproducibility of the endometrial receptivity array is superior to histology as a diagnostic method for endometrial receptivity. Fertil. Steril..

[202] Ruiz-Alonso M., Blesa D., Díaz-Gimeno P., Gómez E., Fernández-Sánchez M., Carranza F., Carrera J., Vilella F., Pellicer A., Simón C. (2013). The endometrial receptivity array for diagnosis and personalized embryo transfer as a treatment for patients with repeated implantation failure. Fertil. Steril..

[203] Bassil R., Casper R., Samara N., Hsieh T.B., Barzilay E., Orvieto R., Haas J. (2018). Does the endometrial receptivity array really provide personalized embryo transfer?. J. Assist. Reprod. Genet..

[204] Enciso M., Carrascosa J.P., Sarasa J., Martínez-Ortiz P.A., Munné S., Horcajadas J.A., Aizpurua J. (2018). Development of a new comprehensive and reliable endometrial receptivity map (ER Map/ER Grade) based on RT-qPCR gene expression analysis. Hum. Reprod..

[205] Croxatto H.B., Ortiz M.E., Diaz S., Hess R., Balmaceda J., Croxato H.D. (1978). Studies on the duration of egg transport by the human oviduct. II. Ovum location at various intervals following luteninizing hormone peak. Am. J. Obstet. Gynecol..

[206] Buster J.E., Bustillo M., Rodi I.A., Cohen S.W., Hamilton M., Simon J.A., Thorneycroft I.H., Marshall J.R. (1985). Biologic and morphologic development of donated human ova recovered by nonsurgical uterine lavage. Am. J. Obstet. Gynecol..

[207] Mossman H.W., Blandau R.J. (1971). Orientation and site of attachment of the blastocyst: A comparative study. Biology of the Blastocyst.

[208] Rasweiler J.J., Badwaik N.K. (1999). Relationships between orientation of the blastocyst during implantation, position of the chorioallantoic placenta, and vascularization of the uterus in the noctilionoid bats Carollia perspicillata and Noctilio sp.. Placenta.

[209] Kirby D.R., Potts D.M., Wilson I.B. (1967). On the orientation of the implanting blastocyst. J. Embryol. Exp. Morphol..

[210] Gardner R.L., Edwards R.G. (1990). Location and orientation of implantation. Establishing a Successful Human Pregnancy.

[211] Rasweiler J.J., Badwaik N.K. (1996). Unusual aspects of inner cell mass formation, endoderm differentiation, Reichert’s membrane development, and amniogenesis in the lesser bulldog bat, Noctilio albiventris. Anat. Rec..

[212] Paria B.C., Ma W., Tan J., Raja S., Das S.K., Dey S.K., Hogan B.L. (2001). Cellular and molecular responses of the uterus to embryo implantation can be elicited by locally applied growth factors. Proc. Natl. Acad. Sci. USA.

[213] Beer A.E., Billingham R.E. (1970). Implantation, transplantation, and epithelial-mesenchymal relationships in the rat uterus. J. Exp. Med..

[214] McLaren A. (1969). Stimulus and response during early pregnancy in the mouse. Nature.

[215] Hetherington C.M. (1968). Induction of deciduomata in the mouse by carbon dioxide. Nature.

[216] Zhang S., Kong S., Wang B., Cheng X., Chen Y., Wu W., Wang Q., Shi J., Zhang Y., Wang S. (2014). Uterine Rbpj is required for embryonic-uterine orientation and decidual remodeling via Notch pathway-independent and -dependent mechanisms. Cell Res..

[217] Goad J., Ko Y.A., Kumar M., Syed S.M., Tanwar P.S. (2017). Differential Wnt signalling activity limits epithelial gland development to the anti-mesometrial side of the mouse uterus. Dev. Biol..

[218] Kimie Y., Rika S., Eriko H., Shunzo K., Yoshihiro K., Ken K., Motonori H., Hitoshi S. (1994). Trypsin-like hatching enzyme of mouse blastocysts: Evidence for its participation in hatching process before zona shedding of embryos. Dev. Growth Differ..

[219] O’Sullivan C.M., Liu S.Y., Karpinka J.B., Rancourt D.E. (2002). Embryonic hatching enzyme strypsin/ISP1 is expressed with ISP2 in endometrial glands during implantation. Mol. Reprod. Dev..

[220] Perona R.M., Wassarman P.M. (1986). Mouse blastocysts hatch in vitro by using a trypsin like proteinase associated with cells of mural trophectoderm. Dev. Biol..

[221] Sireesha G.V., Mason R.W., Hassanein M., Tonack S., Navarrete Santos A., Fischer B., Seshagiri P.B. (2008). Role of cathepsins in blastocyst hatching in the golden hamster. Mol. Hum. Reprod..

[222] Mishra A., Seshagiri P.B. (2000). Evidence for the involvement of species-specific embryonic protease in zona dissolution of hamster blastocysts. Mol. Hum. Reprod..

[223] Piccirilli D., Baldini E., Massimiani M., Camaioni A., Salustri A., Bernardini R., Centanni M., Ulisse S., Moretti C., Campagnolo L. (2018). Thyroid hormone regulates protease expression and activation of Notch signaling in implantation and embryo development. J. Endocrinol..

[224] Dickinson D.P. (2002). Cysteine peptidases of mammals: Their biological roles and potential effects in the oral cavity and other tissues in health and disease. Crit. Rev. Oral. Biol. Med..

[225] Afonso S., Romagnano L., Babiarz B. (1997). The expression and function of cystatin C and cathepsin B and cathepsin L during mouse embryo implantation and placentation. Development.

[226] O’Sullivan C.M., Liu S.Y., Rancourt S.L., Rancourt D.E. (2001). Regulation of the strypsinrelated proteinase ISP2 by progesterone in endometrial gland epithelium during implantation in mice. Reproduction.

[227] Sharma N., Kumar R., Renaux B., Saifeddine M., Nishikawa S., Mihara K., Ramachandran R., Hollenberg M.D., Rancourt D.E. (2011). Implantation serine proteinase 1 exhibits mixed substrate specificity that silences signaling via proteinase activated receptors. PLoS ONE.

[228] Sharma N., Liu S., Tang L., Irwin J., Meng G., Rancourt D.E. (2006). Implantation Serine Proteinases heterodimerize and are critical in hatching and implantation. BMC Dev. Biol..

[229] O’Sullivan C.M., Tang L., Xu H., Liu S., Rancourt D.E. (2004). Origin of the murine implantation serine proteinase subfamily. Mol. Reprod. Dev..

[230] Lindenberg S. (1991). Experimental studies on the initial trophoblast endometrial interaction. Dan. Med. Bull..

[231] Hertig A.T., Rock J., Adams E.C. (1956). A description of 34 human ova within the first 17 days of development. Am. J. Anat..

[232] Sharma A., Kumar P. (2012). Understanding implantation window, a crucial phenomenon. J. Hum. Reprod. Sci..

[233] Bischof P., Campana A. (1996). A model for implantation of the human blastocyst and early placentation. Hum. Reprod. Update.

[234] Denker H.W. (1993). Implantation: A cell biological paradox. J. Exp. Zool..

[235] Cheng J.G., Chen J.R., Hernandez L., Alvord W.G., Stewart C.L. (2001). Dual control of LIF expression and LIF receptor function regulate Stat3 activation at the onset of uterine receptivity and embryo implantation. Proc. Natl. Acad. Sci. USA.

[236] Catalano R., Johnson M.H., Campbell E.A., Charnock-Jones D.S., Smith S.K., Sharkey A.M. (2005). Inhibition of Stat3 activation in the endometrium prevents implantation: A nonsteroidal approach to contraception. Proc. Natl. Acad. Sci. USA.

[237] Song H., Lim H., Das S.K., Paria B.C., Dey S.K. (2000). Dysregulation of EGF family of growth factors and COX-2 in the uterus during the preattachment and attachment reactions of the blastocyst with the luminal epithelium correlates with implantation failure in LIF-deficient mice. Mol. Endocrinol..

[238] Pawar S., Starosvetsky E., Orvis G.D., Behringer R.R., Bagchi I.C., Bagchi M.K. (2013). STAT3 regulates uterine epithelial remodeling and epithelial-stromal crosstalk during implantation. Mol. Endocrinol..

[239] Hantak A.M., Bagchi I.C., Bagchi M.K. (2014). Role of uterine stromal-epithelial crosstalk in embryo implantation. Int. J. Dev. Biol..

[240] Laird S.M., Tuckerman E.M., Dalton C.F., Dunphy B.C., Li T.C., Zhang X. (1997). The production of leukaemia inhibitory factor by human endometrium: Presence in uterine flushings and production by cells in culture. Hum. Reprod..

[241] Hambartsoumian E. (1998). Endometrial leukemia inhibitory factor (LIF) as a possible cause of unexplained infertility and multiple failures of implantation. Am. J. Reprod. Immunol..

[242] Srisuparp S., Strakova Z., Fazleabas A.T. (2001). The role of chorionic gonadotropin (CG) in blastocyst implantation. Arch. Med. Res..

[243] Alfthan H., Stenman U.H. (1996). Pathophysiological importance of various molecular forms of human choriogonadotropin. Mol. Cell. Endocrinol..

[244] Aplin J.D., Spanswick C., Behzad F., Kimber S.J., Vicovac L. (1996). Integrins beta 5, beta 3 and alpha v are apically distributed in endometrial epithelium. Mol. Hum. Reprod..

[245] Apparao K.B., Murray M.J., Fritz M.A., Meyer W.R., Chambers A.F., Truong P.R., Lessey B.A. (2001). Osteopontin and its receptor alphavbeta(3) integrin are coexpressed in the human endometrium during the menstrual cycle but regulated differentially. J. Clin. Endocrinol. Metab..

[246] Reddy K.V., Mangale S.S. (2003). Integrin receptors: The dynamic modulators of endometrial function. Tissue Cell.

[247] Genbacev O.D., Prakobphol A., Foulk R.A., Krtolica A.R., Ilic D., Singer M.S., Yang Z.Q., Kiessling L.L., Rosen S.D., Fisher S.J. (2003). Trophoblast L-selectin-mediated adhesion at the maternal-fetal interface. Science.

[248] Foulk R.A., Zdravkovic T., Genbacev O., Prakobphol A. (2007). Expression of L-selectin ligand MECA-79 as a predictive marker of human uterine receptivity. J. Assist. Reprod. Genet..

[249] Carson D.D., Julian J., Lessey B.A., Prakobphol A., Fisher S.J. (2006). MUC1 is a scaffold for selectin ligands in the human uterus. Front. Biosci..

[250] Rowlands T.M., Symonds J.M., Farookhi R., Blaschuk O.W. (2000). Cadherins: Crucial regulators of structure and function in reproductive tissues. Rev. Reprod..

[251] Shih I.M., Hsu M.Y., Oldt R.J., Herlyn M., Gearhart J.D., Kurman R.J. (2002). The Role of E-cadherin in the Motility and Invasion of Implantation Site Intermediate Trophoblast. Placenta.

[252] MacCalman C.D., Furth E.E., Omigbodun A., Bronner M., Coutifaris C., Strauss J.F. (1996). Regulated expression of cadherin-11 in human epithelial cells: A role for cadherin-11 in trophoblast-endometrium interactions?. Dev. Dyn..

[253] Chillaron J., Roca R., Valencia A., Zorzano A., Palacin M. (2001). Heteromeric amino acid transporters: Biochemistry, genetics, and physiology. Am. J. Physiol. Renal Physiol..

[254] Tsurudome M., Ito Y. (2000). Function of fusion regulatory proteins (FRPs) in immune cells and virus-infected cells. Crit. Rev. Immunol..

[255] Dominguez F., Simon C., Quinonero A., Ramirez M.A., Gonzalez-Munoz E., Burghardt H., Cervero A., Martinez S., Pellicer A., Palacin M. (2010). Human endometrial CD98 is essential for blastocyst adhesion. PLoS ONE.

[256] Cuman C., Menkhorst E.M., Rombauts L.J., Holden S., Webster D., Bilandzic M., Osianlis T., Dimitriadis E. (2013). Preimplantation human blastocysts release factors that differentially alter human endometrial epithelial cell adhesion and gene expression relative to IVF success. Hum. Reprod..

[257] Garcia-Lloret M., Morrish D.W., Guilbert L.J. Functional expression of CSF-1 receptors on normal human trophoblast. Proceedings of the Third European Placental Group Meeting.

[258] Haimovici F., Anderson D.J. (1993). Cytokines and growth factors in implantation. Microsc. Res. Tech..

[259] Pollard J.W., Hunt J.S., Wiktor-Jedrzejczak W., Stanley E.R. (1991). A pregnancy defect in the osteopetrotic (opop) mouse demonstrates the requirement for CSF-1 in female fertility. Dev. Biol..

[260] Pijnenborg R., Bland J.M., Robertson W.B., Dixon G., Brosens I. (1981). The pattern of interstitial trophoblastic invasion of the myometrium in early human pregnancy. Placenta.

[261] Giudice L.C. (1999). Potential biochemical markers of uterine receptivity. Hum. Reprod..

[262] Burrows T.D., King A., Loke Y. (1996). Trophoblast migration during human placental implantation. Hum. Reprod. Update.

[263] Pijnenborg R., Robertson W.B., Brosens I., Dixon G. (1981). Trophoblast invasion and establishment of haemochorial placentation in man and laboratory animals. Placenta.

[264] Hunkapiller N.M., Gasperowicz M., Kapidzic M., Plaks V., Maltepe E., Kitajewski J., Cross J.C., Fisher S. (2011). A role for Notch signaling in trophoblast endovascular invasion and in the pathogenesis of pre-eclampsia. Development.

[265] Shimonovitz S., Hurwitz A., Dushnik M., Anteby E., GevaEldar T., Yagel S. (1994). Developmental regulation of the expression of 72 and 92 kd type IV collagenases in human trophoblasts: A possible mechanism for control of trophoblast invasion. Am. J. Obstet. Gynecol..

[266] Cañete-Soler R., Gui Y.H., Linask K.K., Muschel R.J. (1995). Developmental expression of MMP-9 (gelatinase B) mRNA in mouse embryos. Dev. Dyn..

[267] Huppertz B., Kertschanska S., Demir A.Y., Frank H.G., Kaufmann P. (1998). Immunohistochemistry of matrix metalloproteinases (MMP), their substrates, and their inhibitors (TIMP) during trophoblast invasion in the human placenta. Cell Tissue Res..

[268] Meisser A., Chardonnens D., Campana A., Bischof P. (1999). Effects of tumour necrosis factor-alpha, interleukin-1 alpha, macrophage colony stimulating factor and transforming growth factor beta on trophoblastic matrix metalloproteinases. Mol. Hum. Reprod..

[269] Bischof P., Meisser A., Campana A., Tseng L. (1998). Effects of deciduaconditioned medium and insulin-like growth factor binding protein-1 on trophoblastic matrix metalloproteinases and their inhibitors. Placenta.

[270] Castellucci M., De Matteis R., Meisser A., Cancello R., Monsurro V., Islami D., Sarzani R., Marzioni D., Cinti S., Bischof P. (2000). Leptin modulates extracellular matrix molecules and metalloproteinases: Possible implications for trophoblast invasion. Mol. Hum. Reprod..

[271] Licht P., Russu V., Wildt L. (2001). On the role of human chorionic gonadotropin (hCG) in the embryo-endometrial microenvironment: Implications for differentiation and implantation. Semin. Reprod. Med..

[272] Qiu Q., Yang M., Tsang B.K., Gruslin A. (2004). EGF-induced trophoblast secretion of MMP-9 and TIMP-1 involves activation of both PI3K and MAPK signaling pathways. Reproduction.

[273] Massimiani M., Vecchione L., Piccirilli D., Spitalieri P., Amati F., Salvi S., Ferrazzani S., Stuhlmann H., Campagnolo L. (2015). Epidermal growth factor-like domain 7 (EGFL7) promotes migration and invasion of human trophoblast cells through activation of MAPK, PI3K and NOTCH signaling pathways. Mol. Hum. Reprod..

[274] Lacko L.A., Massimiani M., Sones J.L., Hurtado R., Salvi S., Ferrazzani S., Davisson R.L., Campagnolo L., Stuhlmann H. (2014). Novel expression of EGFL7 in placental trophoblast and endothelial cells and its implication in preeclampsia. Mech. Dev..

[275] Taki A., Abe M., Komaki M., Oku K., Iseki S., Mizutani S., Morita I. (2012). Expression of angiogenesis-related factors and inflammatory cytokines in placenta and umbilical vessels in pregnancies with preeclampsia and chorioamnionitis/funisitis. Congenit. Anom..

[276] Meng T., Chen H., Sun M., Wang H., Zhao G., Wang X. (2012). Identification of differential gene expression profiles in placentas from preeclamptic pregnancies versus normal pregnancies by DNA microarrays. OMICS.

[277] Sahin Z., Acar N., Ozbey O., Ustunel I., Demir R. (2011). Distribution of Notch family proteins in intrauterine growth restriction and hypertension complicated human term placentas. Acta Histochem..

[278] Løset M., Mundal S.B., Johnson M.P., Fenstad M.H., Freed K.A., Lian I.A., Eide I.P., Bjørge L., Blangero J., Moses E.K. (2011). A transcriptional profile of the decidua in preeclampsia. Am. J. Obstet. Gynecol..

[279] Sitras V., Paulssen R.H., Grønaas H., Leirvik J., Hanssen T.A., Vårtun A., Acharya G. (2009). Differential placental gene expression in severe preeclampsia. Placenta.

[280] Cobellis L., Mastrogiacomo A., Federico E., Schettino M.T., De Falco M., Manente L., Coppola G., Torella M., Colacurci N., De Luca A. (2007). Distribution of Notch protein members in normal and preeclampsia complicated placentas. Cell Tissue Res..

[281] Massimiani M., Lacko L.A., Burke Swanson C.S., Salvi S., Argueta L.B., Moresi S., Ferrazzani S., Gelber S.E., Baergen R.N., Toschi N. (2019). Increased circulating levels of Epidermal Growth Factor-like Domain 7 in pregnant women affected by preeclampsia. Transl. Res..

[282] Fournier T. (2016). Human chorionic gonadotropin: Different glycoforms and biological activity depending on its source of production. Ann. Endocrinol..

[283] Cole L.A. (2012). hCG, the wonder of today’s science. Reprod. Biol. Endocrinol..

[284] Roth I., Fisher S. (1999). IL-10 is an autocrine inhibitor of human placental cytotrophoblast MMP-9 production and invasion. Dev. Biol..

[285] Higuchi T., Kanzaki H., Nakayama H., Fujimoto M., Hatayama H., Kojima K., Iwai M., Mori T., Fujita J. (1995). Induction of tissue inhibitor of metalloproteinase 3 gene expression during in vitro decidualization of human endometrial stromal cells. Endocrinology.

[286] Reponen P., Leivo I., Sahlberg C., Apte S.S., Olsen B.R., Thesleff I., Tryggvason K. (1995). 92-kDa type IV collagenase and TIMP-3, but not 72-kDa type IV collagenase or TIMP-1 or TIMP-2, are highly expressed during mouse embryo implantation. Dev. Dyn..

[287] Aflalo E.D., Sod-Moriah U.A., Potashnik G., Har-Vardi I. (2004). Differences in the implantation rates of rat embryos developed in vivo and in vitro: Possible role for plasminogen activators. Fertil. Steril..

[288] Schatz F., Aigner S., Papp C., Toth-Pal E., Hausknecht V., Lockwood C.J. (1995). Plasminogen activator activity during decidualization of human endometrial stromal cells is regulated by plasminogen activator inhibitor 1. J. Clin. Endocrinol. Metab..

[289] Simón C., Gimeno M.J., Mercader A., Francés A., Garcia Velasco J., Remohí J., Polan M.L., Pellicer A. (1996). Cytokines-adhesion molecules-invasive proteinases. The missing paracrine/autocrine link in embryonic implantation?. Mol. Hum. Reprod..

[290] Karmakar S., Das C. (2002). Regulation of trophoblast invasion by IL-1β and TGF-β1. Am. J. Reprod. Immunol..

[291] Iacob D., Cai J., Tsonis M., Babwah A., Chakraborty C., Bhattacharjee R.N., Lala P.K. (2008). Decorin-mediated inhibition of proliferation and migration of the human trophoblast via different tyrosine kinase receptors. Endocrinology.

[292] Aplin J.D., Haigh T., Lacey H., Chen C.P., Jones C.J. (2000). Tissue interactions in the control of trophoblast invasion. J. Reprod. Fertil. Suppl..

[293] Su R.W., Strug M.R., Joshi N.R., Jeong J.W., Miele L., Lessey B.A., Young S.L., Fazleabas A.T. (2015). Decreased Notch pathway signaling in the endometrium of women with endometriosis impairs decidualization. J. Clin. Endocrinol. Metab..

[294] Majumdar G., Majumdar A., Verma I.C., Upadhyaya K.C. (2017). Relationship between morphology, euploidy and implantation potential of cleavage and blastocyst stage embryos. J. Hum. Reprod. Sci..

[295] Gardner D.K., Balaban B. (2016). Assessment of human embryo development using morphological criteria in an era of time-lapse, algorithms and ‘OMICS’: Is looking good still important?. Mol. Hum. Reprod..

[296] ALPHA Scientists In Reproductive Medicine, ESHRE Special Interest Group Embryology (2011). Istanbul consensus workshop on embryo assessment: Proceedings of an expert meeting. Reprod. Biomed. Online.

[297] Seli E., Sakkas D., Scott R., Kwok S.C., Rosendahl S.M., Burns D.H. (2007). Noninvasive metabolomic profiling of embryo culture media using Raman and near-infrared spectroscopy correlates with reproductive potential of embryos in women undergoing in vitro fertilization. Fertil. Steril..

[298] Scott R., Seli E., Miller K., Sakkas D., Scott K., Burns D.H. (2008). Noninvasive metabolomic profiling of human embryo culture media using Raman spectroscopy predicts embryonic reproductive potential: A prospective blinded pilot study. Fertil. Steril..

[299] Vergouw C.G., Botros L.L., Roos P., Lens J.W., Schats R., Hompes P.G.A., Burns D.H., Lambalk C.B. (2008). Metabolomic profiling by near-infrared spectroscopy as a tool to assess embryo viability: A novel, non-invasive method for embryo selection. Hum. Reprod..

[300] Krisher R.L., Schoolcraft W.B., Katz-Jaffe M.G. (2015). Omics as a window to view embryo viability. Fertil. Steril..

[301] Ntostis P., Kokkali G., Iles D., Huntriss J., Tzetis M., Picton H., Pantos K., Miller D. (2019). Can trophectoderm RNA analysis predict human blastocyst competency?. Syst. Biol. Reprod. Med..

[302] Kirkegaard K., Villesen P., Jensen J.M., Hindkjær J.J., Kølvraa S., Ingerslev H.J., Lykke-Hartmann K. (2015). Distinct differences in global gene expression profiles in non-implanted blastocysts and blastocysts resulting in live birth. Gene.

[303] Jones G.M., Cram D.S., Song B., Kokkali G., Pantos K., Trounson A.O. (2008). Novel strategy with potential to identify developmentally competent IVF blastocysts. Hum. Reprod..

[304] Weimar C.H., Kavelaars A., Brosens J.J., Gellersen B., de Vreeden-Elbertse J.M., Heijnen C.J., Macklon N.S. (2012). Endometrial stromal cells of women with recurrent miscarriage fail to discriminate between high- and low-quality human embryos. PLoS ONE.

[305] Brosens J.J., Salker M.S., Teklenburg G., Nautiyal J., Salter S., Lucas E.S., Steel J.H., Christian M., Chan Y.W., Boomsma C.M. (2014). Uterine selection of human embryos at implantation. Sci. Rep..

